# Mn Oxide Nanowire/ZIF-8 Composites with Multiple Enzyme-like Activities for Enantioselective Glutamate Sensing

**DOI:** 10.3390/bios15120771

**Published:** 2025-11-25

**Authors:** Guo-Ying Chen, Mao-Ling Luo, Jing-Jing Dai, Feng-Qing Yang

**Affiliations:** Department of Pharmaceutical Engineering, School of Chemistry and Chemical Engineering, Chongqing University, Chongqing 401331, China; 20221801017@stu.cqu.edu.cn (G.-Y.C.); 201718021124@cqu.edu.cn (M.-L.L.); 202018021119@cqu.edu.cn (J.-J.D.)

**Keywords:** tandem enzymes, multiple enzyme-like activities, chiral recognition, ZIF-8, Mn oxide nanowires

## Abstract

A composite material of Mn oxide nanowires and ZIF-8 (Mn_x_O_y_ NWs@ZIF-8-RD) with controllable sizes and morphologies (rhombic dodecahedron-shape) was successfully synthesized under mild reaction conditions. The systematic investigation into the effects of synthesis conditions of the material on their microstructure and crystalline morphology was conducted. The material function as “tandem enzymes”, exhibiting multiple enzyme-like activities, such as peroxidase (POD)- and glutamate-oxidase (Glu OXD)-like activities. Kinetic studies reveal that the Mn_x_O_y_ NWs@ZIF-8-RD has excellent enzyme-like catalytic activity, with high substrate affinity and a maximum reaction rate of (H_2_O_2_: 840.52 × 10^−8^ M·S^−1^). Mn_x_O_y_ NWs@ZIF-8-RD shows remarkable enantioselectivity for Glu enantiomers based on its POD- and Glu OXD-like activities. By integrating theoretical and experimental approaches, the recognition mechanism was preliminarily elucidated. In short, this study offered valuable insights for developing sophisticated functional materials and provided methodological references for Glu enantiomer recognition and quantitative detection.

## 1. Introduction

Amino acids (AAs) serve as the fundamental building blocks of proteins and other biological macromolecules, and their enantiomers often exhibit distinct physiological activities and biological functions [1]. L-AAs can be absorbed and utilized by the human body for protein synthesis, whereas D-amino acids generally lack this bioavailability. Alterations in the expression levels of specific chiral AAs in biological systems are often associated with the early stages of various diseases, such as chronic kidney disease, Alzheimer’s disease, and cancer [2]. For example, L-glutamic acid (L-Glu) plays a critical role in the central nervous system, contributing significantly to the formation and stability of brain functions and participating in cellular energy metabolism [3]. Although D-glutamic acid (D-Glu) is widely distributed in bacterial cell walls, various plants, and in the liver, kidney, and brain tissues of rats, among other biological systems, its functions remain not fully understood [4]. D-Glu cannot be metabolized by living systems, potentially having adverse effects on life, leading to malnutrition and low product safety when present in food and pharmaceuticals [5]. Therefore, accurately distinguishing between Glu enantiomers is of great significance.

Currently, different methods have been developed for the identification and detection of AA enantiomers, such as high-performance liquid chromatography, gas chromatography [6], capillary electrophoresis [7], circular dichroism spectroscopy [8], electrochemical methods [9], and fluorescence-based techniques [5]. Among these methods, fluorescence spectroscopy has emerged as an attractive method due to its operational simplicity, rapid response, low cost, and ease of miniaturization [10]. Nevertheless, the design of chiral binding/reactive sites in fluorescent probes is crucial, typically demanding complex and precise chemical synthesis to achieve stereochemical interactions between the analyte and the probe molecule [10]. Moreover, such fluorescent probes are rarely employed for the enantioselective and chemoselective recognition of specific AAs, and single fluorescent probes are susceptible to interference from other substances, resulting in poor selectivity and interference-resistance capability. Therefore, it is imperative to develop a universal fluorescence sensing strategy for the broad detection of chiral AAs, particularly enabling enantioselective and chemoselective detection of specific amino acids.

Natural enzymes are high-performance biocatalysts characterized by high substrate specificity and selectively catalytic activity. For example, L- and D-amino acid oxidases exhibit broad enantioselectivity toward various AAs, catalyzing their conversion into α-keto acids and H_2_O_2_. However, natural enzymes are often limited by poor stability, difficult recovery, and high cost [11]. In contrast, nanozymes, nanomaterials with enzyme-like activities, offer advantages such as facile synthesis, tunable properties, and enhanced stability. Typical nanozymes include metal oxides, carbon dots, and metal–organic frameworks [12]. Among them, ZIF-8 is particularly attractive due to its straightforward synthesis, high surface area, and size tunability, making it a suitable matrix for constructing multifunctional enzyme-mimetic composites [13]. Manganese (Mn), a multivalent transition metal, has been engineered into diverse nanostructures, such as nanoparticles, nanosheets, and nanowires, many of which exhibit excellent enzyme-like properties [14]. For instance, Wang et al. [15] developed manganese urea nanozymes via a hydrothermal approach, which demonstrated well-defined cubic morphology, crystalline structure, and oxidase-like activity for sensitive detection of As(V). Notably, manganese oxide nanowires (Mn_x_O_y_ NWs) possess a high surface-to-volume ratio that affords abundant active sites, leading to superior catalytic performance. Song et al. [16] reported that MnO_2_ NWs exhibited outstanding electrochemical properties in supercapacitors, including high specific capacitance and cycling stability, attributed to their large specific surface area. Moreover, Mn_x_O_y_ NWs display strong metal and support interactions and good thermal stability, allowing further catalytic enhancement of composite structures. Based on these merits, the composite material combining ZIF-8 and Mn_x_O_y_ NWs is expected to leverage their complementary features and enzyme-like activities, designed as a fluorescence-sensing platform for the enantioselective recognition of AAs.

**Figure 1:**
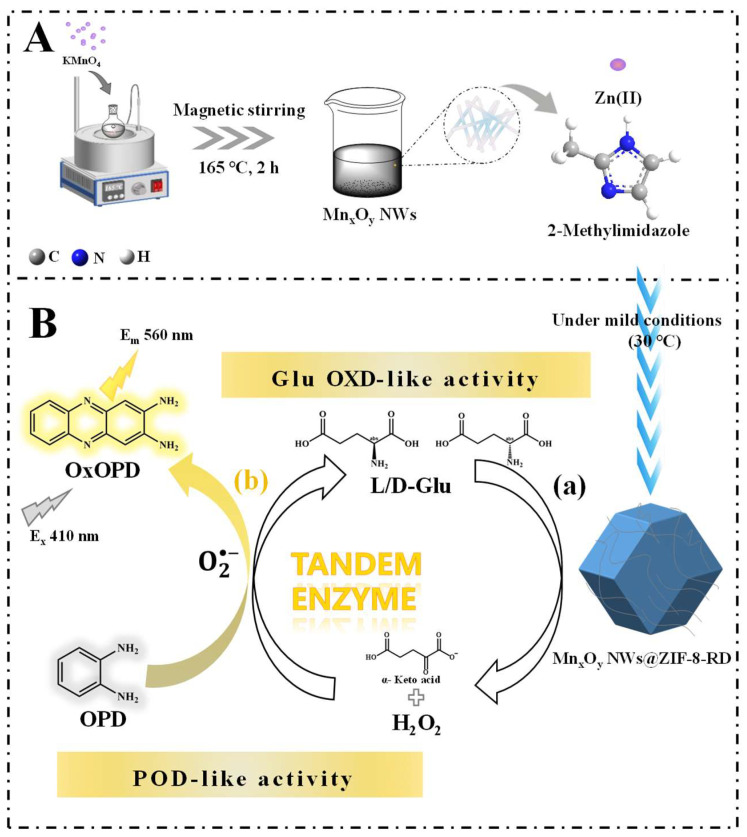
Schematic illustration of materials synthesis process (**A**), the mechanism of Glu enantiomer recognition (**B**). In subfigure (**B**), catalysis of Glu based on Glu OXD-like activity (**a**), and catalysis of H_2_O_2_ and OPD based on the POD-like activity (**b**) of Mn_x_O_y_ NWs@ZIF-8-RD.

Therefore, the composite material of Mn_x_O_y_ NWs@ZIF-8-rhombic dodecahedron (RD) was synthesized under mild conditions in this study (Figure 1). This composite material combining ZIF-8 with Mn_x_O_y_ NWs may fully exploit the synergistic effects of both components, thereby markedly improving the catalytic performance of the material. By incorporating Mn_x_O_y_ NWs into the precursor solution of ZIF-8 and conducting the reaction in an oven set at 30 °C, composite material with specific sizes and shapes can be synthesized (Figure 1A). The conditions for controlling its size and morphology were studied through adjusting the precursor concentration (Mn_x_O_y_ NWs concentration and metal-to-ligand ratio), synthesis temperature and time. Then, the steady-state kinetics of enzyme-like activity (peroxidase (POD) was investigated. Leveraging the Glu OXD (Figure 1B(a)) and POD-like (Figure 1B(b)) activities of Mn_x_O_y_ NWs@ZIF-8-RD, the quantitative detection and chiral recognition of Glu enantiomers can be achieved (Figure 1B). Furthermore, the sensitivity, selectivity, and interference resistance of the developed methods for Glu enantiomers recognition was comprehensively assessed. In addition, through integrating experimental results with density functional theory calculations (DFT), the mechanism of Glu enantioselective recognition was preliminary elucidated. The results of this work not only provide a reference method for the modification of ZIFs, but also proposes novel methods for specific recognition of Glu enantiomers.

## 2. Materials and Methods

### 2.1. Materials and Reagents

Zinc acetate dihydrate (C_4_H_6_O_4_Zn·2H_2_O, AR), D-glutamic acid (D-Glu, 98%), D(+)-arginine (D-Arg, 98%) were obtained from Macklin Co., Ltd. (Shanghai, China). 2-Methylimidazole (2-Hmim, AR), L-glutamic acid (L-Glu, 99%), L-asparagine (L-Asn, BR) were obtained from Aladdin Co., Ltd. (Shanghai, China). Rabbit plasma (with sodium citrate as the anticoagulant), polyvinylpyrrolidone (PVP, AR), L-aspartic acid (L-Asp, 99%) were purchased from Shanghai YuanYe Biological Technology Co., Ltd. (Shanghai, China). Polyethylene glycol (EG, AR), methanol (AR), *o*-phenylenediamine (OPD, 99%) were purchased from Kelong Reagent Co., Ltd. (Chengdu, China). Potassium permanganate (KMnO_4_, 99%) was purchased from Shangxiwei Kang Medical Co., Ltd. (Shanghai, China). L-Methionine (L-Met, 98%) was obtained from Energy Chemical Co., Ltd. (Shanghai, China). D-Methionine (D-Met, 99%), D-aspartic acid (D-Asp, 99%), D-alanine (D-Ala, 98%), D-proline (D-Pro, 99%), D-threonine (D-Thr, 99%) were obtained from Adamas Co., Ltd. (Shanghai, China). L(+)-Arginine (L-Arg, BR) and D-histidine (D-His, 98%), L-lysine (L-Lys, BR), and L-tyrosine (L-Tyr, AR) were purchased from Hua Xia Chemical Co., Ltd. (Chengdu, China). D-Asparagine (D-Asn, 99%), L-leucine (L-Leu, 99%), D-tyrosine (D-Tyr, 99%), and D-leucine (D-Leu, 99%) were obtained from Xinsen Bio Technology Co., Ltd. (Shenzhen, China). L-Alanine (L-Ala, BR) was obtained from Rhawn Chemical Co., Ltd. (Shanghai, China). L-Glutamine (L-Gln, ≥98.5%) was purchased from Notlas Co., Ltd. (Beijing, China). D-Glutamine (D-Gln, 99%), D-isoleucine (D-Ile, 99%), L-threonine (D-Thr, 99%) were obtained from Mreda Technology Co., Ltd. (Beijing, China). L-isoleucine (L-Ile, 99%), L-valine (L-Val, 99%), D-valine (D-Val, 98%+), and D-tryptophan (D-Typ, 99%) were obtained from Meryer Biochemical Co., Ltd. (Shanghai, China). L-Proline (L-Pro, 99%) was obtained from Biotopped Technology Co., Ltd. (Beijing, China). L-Serine (L-Ser, 99%) was purchased from Energy Chemical Co., Ltd. (Shanghai, China). D-Serine (L-Ser, 99%) and D-cysteine (D-Cys, 99%) were obtained from Heowns Bio Technology Co., Ltd. (Tianjin, China). L-Cysteine (L-Cys, 99%) was obtained from Kwangfu Fine Chemical Co., Ltd. (Tianjin, China). D-Lysine (D-Lys, 98%) was purchased from Bide Pharmatech Co., Ltd. (Shanghai, China). L-Phenyalanine (L-Phe, 99.5%) was obtained from Sangon Biotech Co., Ltd. (Shanghai, China). D-Phenyalanine (D-Phe, BR) was obtained from Sinopharm Chemical Co., Ltd. (Shanghai, China).

### 2.2. Instruments

Fluorescence analysis was carried out using an F97Pro uorescence spectrophotometer (Lengguang Technology, Shanghai, China). The environmental scanning electron microscope (ESEM) (Quattro S, Thermo Fisher Scientific, Waltham, MA, USA) was used to characterize the synthesized materials. The Fourier-transform infrared (FT-IR) spectra were recorded using a Nicolet iS50 (Thermo Scientific Inc., Waltham, MA, USA). The sample’s X-ray diffraction (XRD) patterns were acquired through an X’ pert Powder diffractometer (Malvern Panalytical Ltd., Almelo, The Netherlands) with secondary beam graphite monochromated Cu Ka radiation. The images and element composition of materials were obtained through transmission electron microscopy (TEM) (Talos F200S, Thermo Fisher Scientific, Prague, Czech Republic). The ultrapure water used throughout this study was purified by a water purification system (ATSelem 1820A, Antesheng Environmental Protection Equipment, Chongqing, China). An FE28 pH meter (Mettler-Toledo Instruments, Shanghai, China) was used for measuring the pH of solutions. The ultrasonic cleaner was purchased from Kunshan Jielimei Ultrasonic Instrument Co., Ltd. (Kunshan, China).

### 2.3. Synthesis of Mn_x_O_y_ NWs

A 15 mL of ethylene glycol (EG) was added into the flask and heated at 165 °C for 15 min. Subsequently, 0.45 g of polyvinylpyrrolidone (PVP) was dissolved thoroughly in 36 mL of EG in a beaker. Thereafter, a 50 μL of NaCl (1 M) and 1.8 mL of KMnO_4_ (1.4 M, refers to the concentration of MnO_4_^−^ used in the synthesis of Mn_x_O_y_ NWs, and subsequent text will use this representation accordingly) solution were sequentially introduced into the beaker and mixed completely. The resulting mixture was then added dropwise into the above flask under continuous stirring, followed by a reaction at 165 °C for 2 h. Subsequently, the reaction mixture was centrifuged at 10,000 rpm (6745× *g*) for 10 min to yield the final product (Mn_x_O_y_ NWs), which was washed three times with methanol. Lastly, the precipitate was re-dispersed in 12 mL of methanol and stored in a refrigerator at 4 °C before use.

### 2.4. Synthesis of Mn_x_O_y_ NWs@ZIF-8-RD

Initially, 0.0274 g of Zn(CH_3_COOH)_2_·2H_2_O (5 mM) and 0.0205 g of 2-methylimidazole (2-Hmim) (10 mM) were dissolved in 25 mL of methanol. Subsequently, 1 mL of the as-synthesized Mn_x_O_y_ NWs (1.4 M) was added to the solution and subjected to ultrasonication for 5 s to ensure uniform dispersion. The resulting solution was incubated at 30 °C for 60 min, followed by washing twice with methanol and centrifuged using a mini centrifuge for 1.5 min each time. Finally, the rhombic dodecahedron-like product was dispersed in 2 mL of methanol before use.

## 3. Results and Discussion

### 3.1. Characterization of Mn_x_O_y_ NWs and Mn_x_O_y_ NWs@ZIF-8-RD

**Figure 2:**
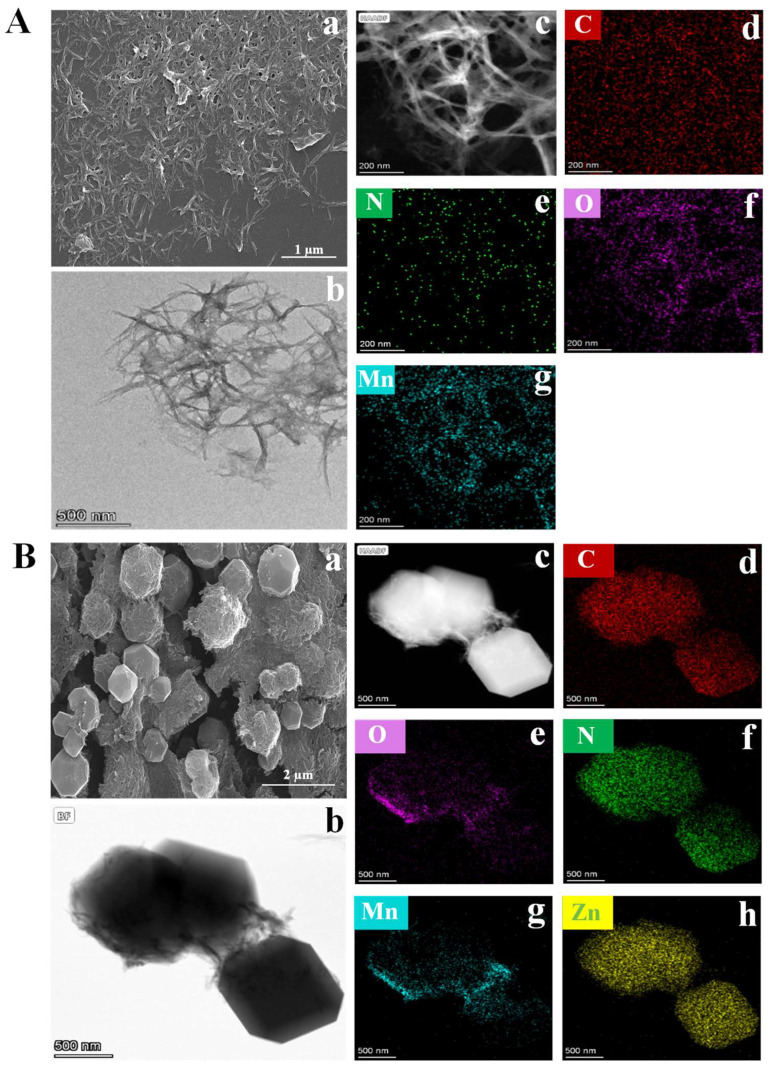
ESEM images (**A**(**a**)), TEM images (**A**(**b**)), and EDX results (**A**(**c**–**g**)) of Mn_x_O_y_ NWs. ESEM images (**B**(**a**)), TEM images (**B**(**b**)), and EDX results (**B**(**c**–**h**)) of Mn_x_O_y_ NWs@ZIF-8-RD.

The successful synthesis of Mn_x_O_y_ NWs was confirmed using ESEM and TEM. As illustrated in Figure 2A(a), Mn_x_O_y_ NWs exhibits the morphology of nanowires with a length of approximately 1 μm. Figure 2A(b–g) show the HAADF-STEM images of Mn_x_O_y_ NWs and the corresponding elemental maps of C (red), N (green), C (red), O (purple), and Mn (blue) (specific distribution of elements is shown in Table S1), which agree with X-ray photoelectron spectroscopy (XPS) analysis results (Figure S1A). As shown in Figure S1B, the C 1s spectra can be deconvoluted into three distinct peaks. The peaks at binding energies of 284.8 eV, 286.2 eV, and 288.4 eV correspond to the C–C/C=C, C–N, and C=O bonds, respectively [17]. The N 1 s spectra can be fitted to C–N C–N (399.52 eV) bond (Figure S1C) [18], which is attributed to the presence of PVP. The O 1s spectra reveals peaks at 529.48 eV, 531.10 eV, and 532.02 eV, corresponding to the Mn–O, C=O, and O–H bonds (Figure S1D) [19], respectively, which indicate that the Mn atoms within the nanowires predominantly form coordination bonds with O atoms and there are adsorbed water on the surface. The Mn 2p spectra consist of two spin–orbit doublet peaks, corresponding to Mn 2p_3/2_ and Mn 2p_1/2_, respectively. The Mn 2p spectra exhibits peaks at 640.75 eV, 652.94 eV, and 645.77 eV, which correspond to the Mn^2+^ and Mn^3+^ oxidation states, as well as satellite peaks, respectively. Additionally, peaks at 642.57 eV and 654.83 eV are corresponding to Mn^4+^ ion (Figure S1E) [19], suggesting a complex oxidation state of Mn. The XPS analysis corroborates the multi-valent nature of Mn within the nanowires, underscoring the complex interplay among different oxidation states. This complexity likely boosts the material’s catalytic efficiency through enabling diverse reaction pathways. Additionally, the presence of PVP implies potential stabilization effects, which may enhance the structural integrity and performance longevity of the nanowires. As depicted in Figure S1F, the FT-IR analysis reveals characteristic peaks at 1640 cm^−1^, 1583 cm^−1^, 1041 cm^−1^, and 512 cm^−1^, which correspond to the C=O/C=C, C–N, C–C, and Mn–O stretching vibrations [18], respectively. These peaks confirm the successful integration of organic and inorganic components, and the strong bonding interactions are pivotal for maintaining the nanowires’ structural stability under operational stresses. These findings align with the XPS data, reinforcing the notion that the nanowires possess a complex surface chemistry that can facilitate the efficient catalytic activity. As shown in Figure S1G, the Mn_x_O_y_ NWs undergo a two-stage thermal decomposition. An initial mass loss of ~12% between 30–220 °C is attributed to the removal of adsorbed and crystalline water/methanol [18]. A subsequent sharp mass loss of 33%, centered around 400 °C with a distinct endothermic peak, corresponds to the oxidative decomposition of organic ligands (PVP and EG), leading to the formation of a stable Mn_x_O_y_ phase [20]. This systematic evolution underscores the material’s structural response to thermal treatment. The gradual loss of organic matter and the formation of a stable Mn_x_O_y_ phase demonstrate the material’s adaptability to extreme conditions. As depicted in Figure S1H, the XRD results reveal the absence of distinct sharp diffraction peaks, indicating an amorphous structure of the sample, which indicates a high degree of disorder within the material. Based on the above observations, the synthesis mechanism of Mn_x_O_y_ NWs can be inferred as follows. KMnO_4_, known for its strong oxidizing properties, undergoes redox reactions when it was added to EG, resulting in the formation of mixed-valence Mn_x_O_y_ compounds (MnO, MnO_2_, Mn_2_O_3_; x = 1, 2; y = 1, 2, 3). These compounds gradually evolve into Mn_x_O_y_ NWs under the influence of surfactant (PVP), which plays a crucial role in facilitating the alignment and uniform growth of the nanowires, thereby ensuring consistent morphology and size [21].

**Figure 3:**
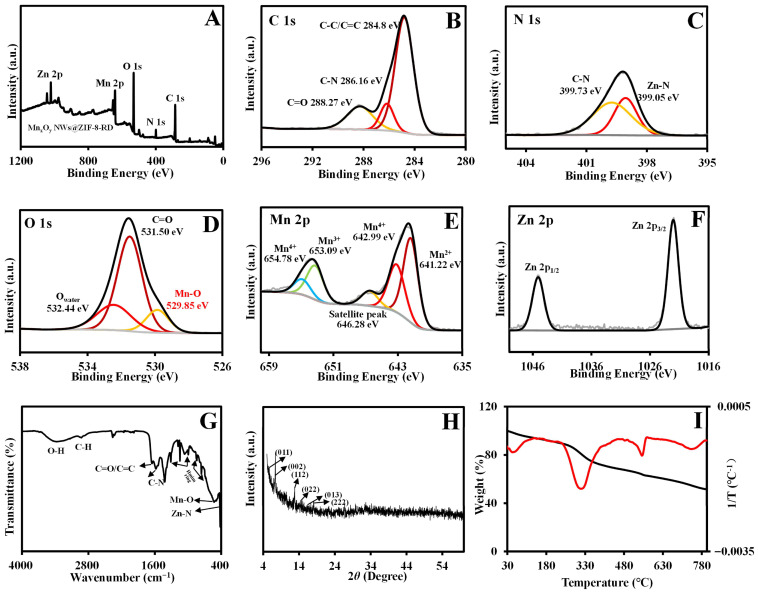
Characterizations of Mn_x_O_y_ NWs@ZIF-8-RD. XPS full spectra (**A**); C 1s (**B**), N 1s (**C**), O 1s (**D**), Mn 2p (**E**), and Zn 2p (**F**) spectra; FT-IR spectra (**G**); XRD results (**H**); TGA results (**I**) of Mn_x_O_y_ NWs@ZIF-8-RD. In subfigure (**I**), the black line represents the weight percentage (%) as a function of temperature, the red line represents the differential heat curve (1/T varies with temperature).

A rhombic dodecahedral composite material of Mn_x_O_y_ NWs@ZIF-8-RD was synthesized in methanol, and its morphology and structure were subsequently analyzed. The ESEM (Figure 2B(a)) and TEM (Figure 2B(b)) images reveal a highly ordered rhombic dodecahedral structure of Mn_x_O_y_ NWs@ZIF-8-RD. The Mn_x_O_y_ NWs are uniformly integrated on both the surface and inside of ZIF-8, retaining its nanowire morphology. The EDX mapping (Figure 2B(c–h)) confirms the uniform distribution of C, N, O, Mn, and Zn elements. Furthermore, the XPS results indicate that the material of Mn_x_O_y_ NWs@ZIF-8-RD possesses the same elemental compositions and chemical bonds as Mn_x_O_y_ NWs (C=O/C=C, C–N, C–C, and Mn–O) and ZIF-8 (C–N, imidazole ring, and Zn–N) (Figure 3A–F) (Relative content (%) of Mn^2+^/Mn^3+^/Mn^4+^: 40.07/18.31/33.72). The FT-IR spectra further confirm the presence of characteristic peaks corresponding to the C=O, C–N, Mn–O, Zn–N, and imidazole rings, validating the composite’s structural integrity (Figure 3G). The XRD results show that the diffraction peaks of Mn_x_O_y_ NWs@ZIF-8-RD closely match with that of ZIF-8, indicating a preserved crystalline structure (Figure 3H). Slightly shifts in peak positions are likely due to the interactions between the nanowires and ZIF-8 framework, which enhance the composite’s stability and functionality. The TGA analysis reveals a gradual weight loss of Mn_x_O_y_ NWs@ZIF-8-RD (Figure 3I), similar to the decomposition of Mn_x_O_y_ NWs. Significant changes occur at 300 °C, corresponding to the removal or degradation of methanol and water molecules (including crystalline water and bound water) within the crystal. As the temperature increases further, the additional weight loss occurred, primarily attributed to the decomposition of organic components such as PVP, EG, and Hmim [22]. Upon heating to 800 °C, the composite’s weight stabilizes at approximately 60% of its initial value.

### 3.2. Optimization of Experimental Conditions for Glu Enantiomers Recognition

**Figure 4:**
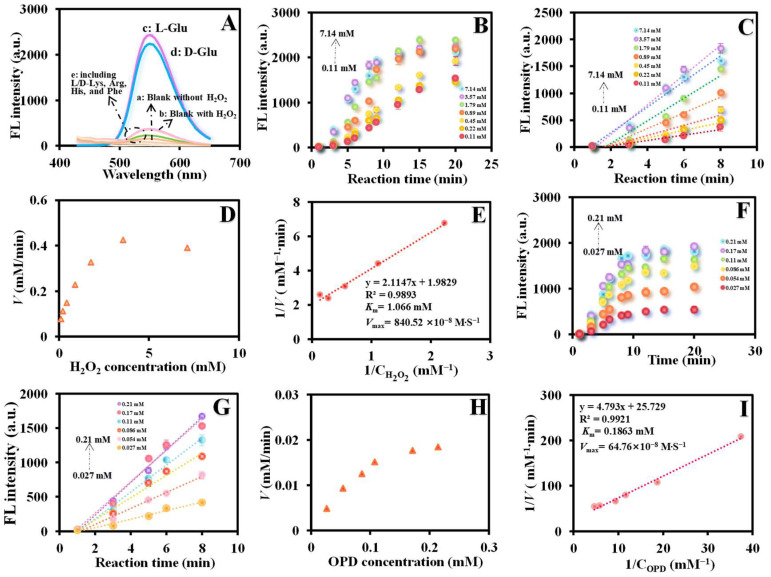
Feasibility analysis of Glu enantiomer recognition (**A**) based on Mn_x_O_y_ NWs@ZIF-8-RD. The relationship between different concentrations of H_2_O_2_ (0.11–7.14 mM) and fluorescence intensity with the reaction time varying from 1 to 20 min (**B**), the linear relationship between fluorescence intensity and reaction time (within 8 min) (**C**), Michaelis–Menten plot (**D**), and Lineweaver–Burk plot (**E**) based on the POD-like activity of Mn_x_O_y_ NWs@ZIF-8-RD. The relationship between different concentrations of OPD (0.027–0.21 mM) and fluorescence intensity with the reaction time varying from 1 to 20 min (**F**), the linear relationship between fluorescence intensity and reaction time (within 8 min) (**G**), Michaelis–Menten plot (**H**), and Lineweaver–Burk plot (**I**) based on the POD-like activity of Mn_x_O_y_ NWs@ZIF-8-RD. In subfigure (**A**), a, Mn_x_O_y_ NWs@ZIF-8-RD + OPD; b, Mn_x_O_y_ NWs@ZIF-8-RD + OPD + H_2_O_2_; c, Mn_x_O_y_ NWs@ZIF-8-RD + L-Glu + OPD; d, Mn_x_O_y_ NWs@ZIF-8-RD + D-Glu + OPD; e, Mn_x_O_y_ NWs@ZIF-8-RD + other AAs (including L/D-Lys, Arg, His, and Phe) + D-Glu.

The Mn_x_O_y_ NWs@ZIF-8-RD can catalyze the oxidation of Glu to produce α-ketoacids and H_2_O_2_ (Glu OXD-like activity), and further catalyze the oxidation of *o*-phenylenediamine (OPD) to ox-OPD (POD like-activity), emitting yellow fluorescence at an excitation wavelength of 410 nm and an emission wavelength of 565 nm (Figure 1B). As shown in Figure 4A, the material of Mn_x_O_y_ NWs@ZIF-8-RD exhibit outstanding POD-like activity, with significant differences in fluorescence intensity observed in the presence (Figure 4A(b)) and absence (Figure 4A(a)) of H_2_O_2_. Additionally, as the addition of amino acid enantiomers (including Glu, Lys, Arg, His, and Phe), these materials demonstrate specific recognition on Glu (Figure 4A(c–e)). And the fluorescence intensity exhibits a significant increase upon the addition of Glu, with certain differences observed between its enantiomers (Figure 4A(c,d)). This selective responsiveness underscores their potential and feasibleness in detecting Glu and its enantiomers.

To further enhance the Glu enantioselective recognition, the effects of the synthetic processes of Mn_x_O_y_ NWs@ZIF-8-RD (Mn_x_O_y_ NWs concentration, Zn(II)/Hmim ratio, synthesis temperature and time) and the reaction conditions (buffer type and pH, reaction temperature, material dilution ratio, OPD concentration, and reaction time) were systematically examined.

#### 3.2.1. The Effect of Mn_x_O_y_ NWs Concentration

The morphology and size of the composite material Mn_x_O_y_ NWs@ZIF-8-RD synthesized using different concentrations of Mn_x_O_y_ NWs exhibits significant concentration dependence. As illustrated in Figure S2A–E, the size of the composite gradually increases from approximately 0.5 μm to 1.1 μm as the concentration of the Mn_x_O_y_ precursor decreased from 1.6 M to 0.8 M, respectively. This trend is further highlighted by the corresponding ESEM images. Specifically, at concentration of 1.6 M (Figure S2A), the composite displays a uniform rhombic dodecahedral structure with the size of about 0.5 μm. At concentrations of 1.4 M (Figure S2B), 1.2 M (Figure S2C), and 1.0 M (Figure S2D), the size of the composite is approximately 1.1 μm, and a rhombic polyhedral structure with Mn_x_O_y_ nanowires growing on its surface can be observed. When the concentration is further reduced to 0.8 M (Figure S2E), the composite maintains an overall rhombic dodecahedral structure, but the ZIF-8 surface exhibits more pronounced mesoporous and macroporous structures, with Mn_x_O_y_ nanoparticles distributed within these pores. These morphological and size variations not only reflect the influence of Mn_x_O_y_ concentration on the growth process of the composite but also reveal its regulatory effect on the ZIF-8 structure, providing an important structural basis for optimizing the preparation process of composite material. The XRD results indicate that as the concentration of the Mn_x_O_y_ precursor is decreased, there is a reduction in the intensity of the characteristic diffraction peaks of ZIF-8, corresponding to the (0 1 1), (1 1 2), (0 2 2), and (2 2 2) crystal planes. Additionally, the broadening of peaks suggests a decrease in crystallinity (Figure S2F). FT-IR spectra further confirms the impact of varying Mn_x_O_y_ precursor concentrations on the structure of Mn_x_O_y_ NWs@ZIF-8-RD. As the concentration is decreased from 1.6 M to 0.8 M, the intensity of ZIF-8’s characteristic absorption peaks diminishes or vanishes (Figure S2G), indicating a disruption to its structural integrity. The absorption peak at 758 cm^−1^ corresponds to the out-of-plane bending vibration of the imidazole ring, and the peaks at 1145 cm^−1^ and 1179 cm^−1^ are attributed to the in-plane bending vibrations. Additionally, the peaks at 1309 cm^−1^ and 1417 cm^−1^ correspond to the stretching vibrations of C–N and C=N bonds, respectively, belonging to the imidazole ring [23]. The characteristic absorption peaks are primarily located at 995 cm^−1^ and 1089 cm^−1^, corresponding to the stretching vibrations of C–C and C–O bonds [24], respectively, which reflect the structural features of Mn_x_O_y_ NWs. As the concentration of Mn_x_O_y_ NWs decreased, the shift and broadening of these peaks suggest a progressive loss of crystallinity and potential phase transformation in ZIF-8. Additionally, the Mn_x_O_y_ NWs@ZIF-8-RD synthesized at different Mn_x_O_y_ concentrations for GOX activity detection and Glu enantiomer recognition was further investigated, respectively. As shown in Figure S2H, considering the morphology and activity of Mn_x_O_y_ NWs@ZIF-8-RD on Glu enantiomers recognition, a Mn_x_O_y_ NWs concentration of 1.4 M was selected as the optimized concentration for subsequent experiments.

#### 3.2.2. The Effect of Zn(II)/Hmim Ratio

The impact of varying molar ratios of Zn(II) and Hmim on the synthesis of Mn_x_O_y_ NWs@ZIF-8-RD (synthesis in methanol) is depicted in Figure S3. When the molar ratio of Zn(II) is less than that of Hmim, Mn_x_O_y_ NWs@ZIF-8-RD predominantly exhibits a rhombic dodecahedral shape with Mn_x_O_y_ NWs growing on the ZIF-8, and the sizes are 0.7 μm (Figure S3A, 1:2), 0.25 μm (Figure S3B, 1:3), and 0.6 μm (Figure S3D, 2:3). In contrast, when the molar ratio of Zn(II) exceeds that of Hmim, the morphology of Mn_x_O_y_ NWs@ZIF-8-RD undergoes a significant transformation, primarily manifesting as a small amount of cloverleaf-like structure (Figure S3C,E,F, 2:1, 3:1, and 3:2, respectively). XRD (Figure S3G) and FT-IR (Figure S3H) results indicate that at the ratios of 1:2, 2:3, and 1:3, Mn_x_O_y_ NWs@ZIF-8-RD retains characteristic diffraction peaks corresponding to the crystal planes of ZIF-8, as well as the vibrational peaks of functional groups, such as the imidazole rings, metal–N/O, and C=C/C=O bonds. The intensity of these peaks is weakened and slightly shifted, likely due to the influence of Mn_x_O_y_ NWs. At the ratios of 2:1, 3:1, and 3:2, the characteristic diffraction peaks of Mn_x_O_y_ NWs@ZIF-8-RD are not prominent in the XRD patterns, while the FT-IR spectra retains some vibrational peaks of the imidazole ring and Mn_x_O_y_ NWs. These findings align with the morphological changes observed by ESEM, suggesting that at these ratios, ZIF-8 crystals are scarce, but the Mn_x_O_y_ NWs and by-products of metal and imidazole ligands are the predominant species. For Glu enantiomers identification (Figure S3I), the fluorescence intensity values varied with changes in the ratio of components Zn(II) to Hmim. After a comprehensive evaluation, a ratio of 1:2 was chosen for further experiments. Therefore, through adjusting the molar ratio of Zn(II) and Hmim, the morphology and size of Mn_x_O_y_ NWs@ZIF-8-RD synthesized in methanol solution can be effectively controlled. Under specific reaction conditions, a lower Zn(II)/Hmim molar ratio is conducive to the formation of larger rhombic dodecahedral structures.

In summary, the optimized conditions for Glu enantioselective recognition are 1.4 M of Mn_x_O_y_ NWs, 1:2 of Zn(II)/Hmim ratio, 30 °C of synthesis temperature, 60 min of synthesis time, aqueous solution (pH 6.5), 55 °C of reaction temperature, 3 of material dilution ratio, 0.27 mM of OPD concentration, and 9 min of reaction time. Among them, the specific analysis of synthesis temperature and time, reaction conditions (buffer type and pH, reaction temperature, material dilution ratio, OPD concentration, and reaction time) can be found in Supplementary Materials.

### 3.3. Enzyme Kinetic

Enzyme kinetics is an effective approach to investigate the progress of enzymatic reactions and the catalytic activity of enzymes [25]. In enzyme kinetics study, the Michaelis–Menten equation holds a central position as it precisely reveals the quantitative relationship between reaction rate (*V*) and substrate concentration ([*S*]), expressed as: *V* = *V*_max_ [*S*]/(*K*_m_ + [*S*]) [26], where *V*_max_ represents the maximum reaction rate; *K*_m_ is the Michaelis constant, indicating the substrate concentration required for the reaction rate to reach half of *V*_max_. *K*_m_ serves as an important measure of the enzyme’s affinity for the substrate, and a lower value indicates a higher affinity between the enzyme and the substrate, and vice versa. The POD-like catalytic activity of Mn_x_O_y_ NWs@ZIF-8-RD was explored, which was evaluated according to the reported method [27]. As depicted in Figure 4B, the fluorescence intensity shows the marked increase with rising H_2_O_2_ concentrations from 0.11 mM to 7.14 mM and increasing reaction time from 1 min to 20 min with good linear relationship within 8 min (Figure 4C). The Michaelis–Menten kinetics (Figure 4D) and its Lineweaver–Burk plot (Figure 4E) for H_2_O_2_ were plotted. The *K*_m_ and *V*_max_ values were calculated to be 1.066 mM and 840.52 × 10^−8^ M/S for H_2_O_2_, respectively. Additionally, fluorescence intensity shows a marked increase with rising OPD concentrations from 0.027 mM to 0.21 mM with good linear relationships within 8 min (Figure 4F,G). The Michaelis–Menten kinetics (Figure 4H) and its Lineweaver–Burk plot (Figure 4I) for OPD reveal the *K*_m_ of 0.1863 mM and *V*_max_ of 64.76 × 10^−8^ M/S. The specific activity (specific activity = overall reaction rate/material mass/ active metal mass/Mn_x_O_y_ mass) and estimated Turnover Frequency (TOF) (TOF = overall reaction rate/moles of active sites) can be calculated to be 1.284 μmol·min^−1^·mg^−1^ (composite material), 24.69 μmol·min^−1^·mg^−1^ (Mn total mass), and 17.30 μmol·min^−1^·mg^−1^ (Mn_x_O_y_ mass), respectively, and TOF of 1.358 min^−1^, demonstrating the enzyme’s high affinity and catalytic efficiency for OPD. In comparison with materials documented in the existing reports (Table 1), the synthesized Mn_x_O_y_ NWs@ZIF-8-RD in this study demonstrates notable catalytic activity towards H_2_O_2_ and OPD, which has significantly lower *K*_m_ value than most analogous materials, suggesting a higher affinity for substrates H_2_O_2_ and OPD. Additionally, its *V*_max_ value is considerably higher, implying a more rapid reaction rate in catalytic processes.

### 3.4. Enantiomeric Differentiation of L/Glu

**Figure 5:**
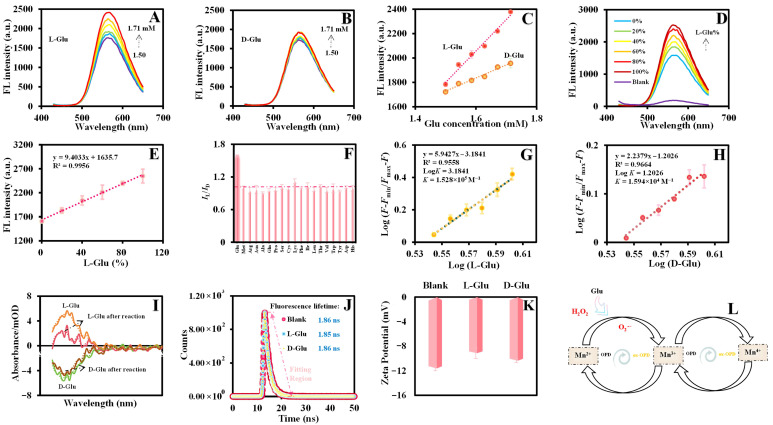
Fluorescence spectra of Mn_x_O_y_ NWs@ZIF-8-RD + OPD in the presence of different concentrations L-Glu (**A**) and D-Glu (**B**) varying from 1.50 to 1.71 mM, and the standard linear relationship between fluorescence intensity and Glu concentrations (**C**). Fluorescence spectra of Mn_x_O_y_ NWs@ZIF-8-RD + OPD in the presence of different enantiomeric percentage of L-Glu (**D**), and the standard linear relationship between fluorescence intensity and enantiomeric percentage of L-Glu (**E**). The influence of different amino acid enantiomers on the reaction solution (**F**). The linear fitting curves between the fluorescence intensity ratio (Log(*F* − *F*_min_)/(*F*_max_ − *F*)) and Log[L-Glu] (**G**) and Log[D-Glu] (**H**). CD spectra (**I**), fluorescence lifetime (**J**), and zeta potential (**K**) results of Mn_x_O_y_ NWs@ZIF-8-RD + OPD with and without L/D-Glu. Catalytic process diagram (**L**) of the oxidation of Glu and OPD by Mn_x_O_y_ NWs@ZIF-8-RD. For subfigures (**A**,**B**), the concentrations of Glu represented by the purple line to the red line are 1.50, 1.54, 1.59, 1.63, 1.67, and 1.71 mM.

As depicted in Figure 5A,B, Mn_x_O_y_ NWs@ZIF-8-RD exhibits Glu enantioselective recognition ability. Upon the addition of a certain concentration (1.50–1.71 mM) of L-Glu to the reaction system, the significant increase in fluorescence intensity can be observed (Figure 5A). In contrast, when the equal concentration of D-Glu is added, there is virtually no change in the fluorescence intensity (Figure 5B). The linear equations are represented as y = 2573.9x − 2060.8 597.91 (R^2^ = 0.9821) (L-Glu) and y = 1073.3x + 118.33 (R^2^ = 0.9765) (D-Glu) (Figure 5C). Furthermore, it is worth emphasizing that the established method not only enables the discrimination of Glu enantiomers within a specific concentration range but also achieves highly selective detection. As exhibited in Figure S7A,B, the fluorescence intensity exhibits the good linear relationships with the concentrations of both L-Glu and D-Glu over a range of 0.27 to 2.14 mM. The corresponding linear equations are y = 1612.9x − 590.97 (R^2^ = 0.9603) (L-Glu) and y = 1426.1x − 479.02 (R^2^ = 0.9808) (D-Glu), and the limits of detection (LOD) was calculated to be 8.29 μM (L-Glu) and 12.76 μM (D-Glu), respectively, using the formula LOD = 3*a*/*k* (*a* represents the calibration deviation from 11 blank experiments, and *k* represents the slope of the calibration curve), which are comparable to other reported methods (Table S2). The results demonstrate that Mn_x_O_y_ NWs@ZIF-8-RD can serve as probes for Glu detection with high sensitivity and also can effectively differentiate Glu enantiomers. Furthermore, through adjusting the percentage of L-Glu in the mixture, the enantiomeric excess recognition ability of Mn_x_O_y_ NWs@ZIF-8-RD was investigated. The experimental results demonstrate that as the proportion of L-Glu increased (0–100%), the fluorescence intensity is gradually increased (Figure 5D), and a good linear relationship is obtained between the fluorescence intensity and L-Glu proportion (Figure 5E) (y = 9.4033x + 1635.7, R^2^ = 0.9956). Therefore, Mn_x_O_y_ NWs@ZIF-8-RD can not only accurately distinguish the enantiomers of Glu but also precisely quantify the enantiomeric excess of L-Glu.

To confirm the specificity of detection method based on Mn_x_O_y_ NWs@ZIF-8-RD, the fluorescence of Mn_x_O_y_ NWs@ZIF-8-RD with 18 kinds of amino acids (Glu, Met, Arg, Asn, Ala, Gln, Pro, Ser, Cys, Lys, Phe, Ile, Thr, Val, Trp, Tyr, Asp, and His) enantiomers under the same conditions was obtained. As shown in Figure 5F, compared with the influence of Glu on the fluorescence intensity (*I*_L_/*I*_D_) of reaction solution (Mn_x_O_y_ NWs@ZIF-8-RD + substrate), the other chiral amino acids show negligible influence, indicating that the fluorescence sensor based on the Mn_x_O_y_ NWs@ZIF-8-RD can effectively achieve the recognition of L/D-Glu (*I*_L_/*I*_D_ = 1.59). Moreover, as exhibited in Figure S7H, the possible presence of metal ions, nucleosides, glucose, etc., in plasma samples can also show negligible effects on the sensor, indicating the good specificity of the method established in this study in identifying Glu enantiomers. Furthermore, the stability of Mn_x_O_y_ NWs@ZIF-8-RD for enantioselective recognition and quantitative detection of Glu was investigated. After storing the materials suspensions at 4 °C for 28 days, the POD-like activities remained above 90% of its initial activities (Figure S7C), which indicates that it possesses good stability. In addition, Mn_x_O_y_ NWs@ZIF-8-RD also exhibits good inter batch stability with RSD < 5% (Figure S7I) and with minor morphological differences (Figure S7J–L). The storage stability and inter batch reproducibility are essential for their applications in enantioselective recognition and quantitative detection to ensure the consistency and reliability of the materials under varying experimental conditions.

To further substantiate the applicability of this method in real sample analysis, spiking and recovery experiments for L/D-Glu in rabbit plasma (rabbit plasma samples were centrifuged and filtered before being diluted 100-fold for analysis) were conducted (according to the linear equations in Figure S7A,B). The experimental outcomes demonstrate that the recovery rates for L/D-Glu in rabbit plasma range from 93.0% to 110.9%, with RSD less than 3.3% (Table S3). These data confirm the method’s accuracy and precision in rabbit plasma analysis. Therefore, the detection method established in this study is well-suited for complex biological sample analysis, offering a reliable technique for the identification of L/D-Glu.

### 3.5. Chiral Recognition Mechanism

To further investigate the mechanism of Mn_x_O_y_ NWs@ZIF-8-RD catalyzing the oxidation of OPD by H_2_O_2_, the radical scavenger experiments were designed and conducted aimed at identifying the active radicals that might be produced during the catalytic process. In the experiments, isopropanol (IPA), ascorbic acid (AA), EDTA-2Na, benzoquinone (BQ), and His were selected as scavengers, which specifically capture hydroxyl radicals (·OH), oxygen, oxygen vacancies (h^+^), superoxide anions ($O_{2}^{•-}$), and singlet oxygen (^1^O_2_), respectively [34]. The experimental results clearly show that the fluorescence intensity significantly decreased upon the addition of BQ (Figure S7D), and this trend continues as the concentrations increased (Figure S7E). This observation compellingly demonstrates that $O_{2}^{•-}$ plays an essential role in the catalysis of H_2_O_2_ oxidation of OPD by Mn_x_O_y_ NWs@ZIF-8-RD. The generation of $O_{2}^{•-}$ was confirmed by electron paramagnetic resonance (EPR) spin-trapping spectroscopy using 5,5-dimethyl-1-pyrroline n-oxide (DMPO). As shown in Figure S7F, the reaction mixture of Mn_x_O_y_ NWs@ZIF-8-RD with H_2_O_2_ and DMPO produced a characteristic six-line EPR signal, which confirmed the presence of $O_{2}^{•-}$, and provided direct evidence for the production of it in this reaction system [35]. Furthermore, the analysis of the high-resolution Mn 2p XPS spectrum (Figure 3E) of Mn_x_O_y_ NWs@ZIF-8-RD revealed two redox couples presented, Mn^3+^/Mn^2+^ and Mn^4+^/Mn^3+^, which endows material with excellent POD-like activity. Thus, it can be inferred that Mn_x_O_y_ NWs@ZIF-8-RD catalyzes the oxidation of Glu to generate *α*-keto acid and H_2_O_2_. A visual bubble assay leveraging a reaction between superoxide dismutase (SOD) and H_2_O_2_ (H_2_O_2_ → O_2_ + 2H_2_O) was confirmed the generation of H_2_O_2_, as evidenced by significant O_2_ bubbles only in the presence of the active material and L-Glu (number of O_2_ bubbles, a > c > b) (Figure S8A). Liquid chromatography-mass spectrometry (LC-MS) analysis further identified the *α*-keto acid (*m*/*z* 145, –H) specifically in the active material reaction system (Figure S8B, (a): L-Glu, (b): Mn_x_O_y_ NWs@ZIF-8-RD + L-Glu, (c): heat-deactivated Mn_x_O_y_ NWs@ZIF-8-RD + L-Glu). The minimal signals in the heat-deactivated control (Figure S8B(c)) confirm the conversion is material-catalyzed, providing direct evidence for the oxidation of L-Glu to *α*-keto acid and H_2_O_2_, thus supporting the proposed cascade mechanism. Subsequently, the generation H_2_O_2_ undergoes further decomposition to generate free radicals ($O_{2}^{•-}$) (Mn^2+^ + H_2_O_2_→Mn^3+^ + $O_{2}^{•-}$ + 2H^+^, Mn^3+^ + H_2_O_2_ → Mn^4+^ + $O_{2}^{•-}$ + 2H^+^), which drive the formation of ox-OPD (Figure S7G and Figure 5L) ($O_{2}^{•-}$ + OPD + H^+^ → ox-OPD + H_2_O). In addition, the reduction of Mn^3+^or Mn^2+^ may directly oxidize OPD to ox-OPD (Mn^4+^ + OPD→Mn^3+^ + ox-OPD, Mn^3+^ + OPD→Mn^2+^ + ox-OPD). In short, the mechanism by which Mn_x_O_y_ NWs@ZIF-8-RD catalyzes the generation of active intermediates from H_2_O_2_ and subsequently oxidizes OPD to form ox-OPD is deduced as above [19,36].

The mechanism of Mn_x_O_y_ NWs@ZIF-8-RD recognizing Glu enantiomers was explored. The exploration of the binding affinity strength between the material and L/D-Glu was conducted utilizing the Hill equation [37] (Equation (1)):

Log (*F* − *F*_min_)/(*F*_max_ − *F*) = Log*K* + *n* Log [L/D-Glu] (1)

where *F*_min_, *F*_max_ and *F* are the emission intensities in the absence, saturated L/D-Glu is present and the given amount of L/D-Glu concentration is added, respectively. *K* represents the binding affinity constant.

The fitting curves obtained by the Hill equation are y = 5.9427x − 3.1841 (L-Glu) (Figure 5G) and y = 2.2379x − 1.2026 (D-Glu) (Figure 5H), and the binding constant *K* of L/D-Glu is estimated to be 1.528 × 10^5^ M^−1^ and 1.594 × 10^4^ M^−1^ respectively, indicating a stronger interaction of L-Glu with the material. Figure 5I illustrates that L/D-Glu displays distinct CD signals in circular dichroism spectroscopy, notably at 205 nm. After introducing Mn_x_O_y_ NWs@ZIF-8-RD and being reacted with L/D-Glu for a set duration, the CD peak intensity of L-Glu was significantly reduced, whereas D-Glu’s CD peak intensity changed marginally. This can be interpreted through the “preferential interaction” theory of enantioselective recognition [38], suggesting that Mn_x_O_y_ NWs@ZIF-8-RD interacts differently with L-Glu and D-Glu. Fluorescence lifetime measurements corroborate these findings, indicating no substantial alteration upon interaction with L/D-Glu (Figure 5J), implying ignorable impact on ox-OPD’s energy or electron transfer processes and mainly due to the difference in the interaction with the material. Zeta potential analysis (Figure 5K) further reveals a marked potential shift upon binding with L-Glu, in contrast to a subtle change with D-Glu, hinting at distinct interactions. Collectively, these results, including the binding constant, CD intensity, and zeta potential difference, and the fluorescence lifetime, validate the differential catalytic efficacy of Mn_x_O_y_ NWs@ZIF-8-RD for L-Glu and D-Glu.

### 3.6. DFT Calculations

**Figure 6:**
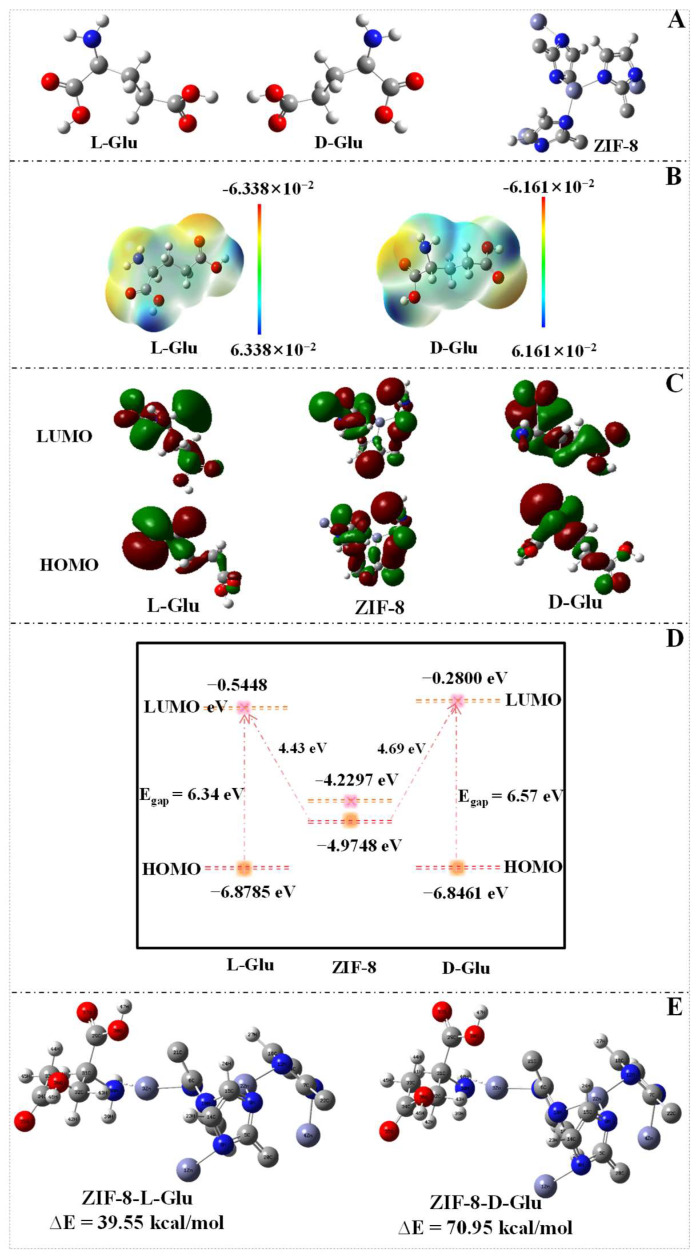
DFT optimized 3D molecular structure of L-Glu, D-Glu, and the structural unit of ZIF-8 (**A**). MEP images of L-Glu and D-Glu (**B**). FMOs of L-Glu, D-Glu, and ZIF-8 (**C**), electron transfer (**D**) between L/D-Glu and ZIF-8, and optimized geometries for the binding energy between ZIF-8 and L/D-Glu (**E**). Noting: the ZIF-8 here refers to the structural unit of it.

To further elucidate the mechanism of the differential effects of Glu enantiomers on the fluorescence intensity of reaction solution, the DFT calculation was utilized to assess molecular conformation, electronic properties, molecular electrostatic potential (MEP), frontier molecular orbitals (FMOs), and interactions between Mn_x_O_y_ NWs@ZIF-8-RD’s primary units and Glu enantiomers. To delve into the characteristics of ZIF-8 as the primary building block of Mn_x_O_y_ NWs@ZIF-8-RD, its precise spatial configuration was obtained from the Cambridge Structural Database (CSD) (Figure S9) and selected an appropriate unit cell for computational modeling. All DFT calculations were performed using the Gaussian 09 software suite, with geometry optimizations carried out using the B3LYP functional and a 6-311++G(d,p) basis set. Initially, the comprehensive geometric optimization and frequency calculations were conducted on L/D-Glu and ZIF-8 to accurately determine and obtain their lowest energy conformations (Figure 6A). The analysis of MEP maps reveals that the electron-rich regions (negative potential, indicated in red) of L-Glu and D-Glu are primarily located near the oxygen of –COOH group and the nitrogen of the –NH_2_ group (Figure 6B). This concentration of electron density is due to the electron-attracting nature of these electronegative atoms, which results in areas of high electron cloud density. These regions are more susceptible to electrophilic attacks [39]. Conversely, the electron-deficient regions (positive potential, indicated in blue) are mainly found near the hydrogen of –COOH and –NH_2_ groups, where the electron cloud density is lower due to their connection with the electronegative atoms, making these areas more prone to nucleophilic attacks [39]. The differences in charge distribution not only reveal the electronic characteristics within the molecules but also provide important clues for understanding their chemical reactivity.

In examining of the electronic structure of materials and molecules, the FMOs theory, which involves the lowest unoccupied molecular orbital (LUMO) and the highest occupied molecular orbital (HOMO), not only offers profound insights into the electron transfer mechanisms and optical properties of materials and molecules but also plays a significant role in predicting donor-acceptor interactions between interacting species [40]. The HOMO-LUMO energy gap (E_gap_) is a crucial parameter for understanding molecular stability and reactivity. Based on this, the following parameters can be calculated to better visualize the reactivity of materials or molecules [41,42]: electronegativity (χ), chemical potential (µ), total hardness (η), total softness (S), absolute softness (σ), and electrophilicity index (ω) (Equations (2)–(8)):

E_gap_ = E_LUMO_ − E_HOMO_ (2)

(3) $$\chi =-\frac{1}{2}\;(E_{\mathrm{LUMO}}\;+\;E_{\mathrm{HOMO}})$$

µ = −χ(4)

(5) $$\eta =-\frac{1}{2}\;(E_{\mathrm{LUMO}}\;-\;E_{\mathrm{HOMO}})$$

(6) $$S=\frac{1}{2\eta }$$

(7) $$\sigma =\frac{1}{\eta }$$

ω = µ^2^/(2η) (8)

The HOMO and LUMO diagrams for L/D-Glu and ZIF-8 are shown in Figure 6C. It is noteworthy that for ZIF-8, the HOMO is primarily concentrated on the imidazole rings, indicating that interactions with electron-deficient compounds such as adsorbed metals mainly occur through the N and H atoms on the imidazole rings. The LUMO is centered on the Zn atoms, suggesting that ZIF-8 can engage in strong interactions with molecules that have electron-donating sites [43]. This electronic distribution characteristic endows ZIF-8 with unique reactivity and selectivity in chemical reactions [44]. As the properties listed in Table S4, by comparing the values between L-Glu and D-Glu, it can be concluded that the L-Glu exhibits superior reactivity and stability compared with the D-Glu, which lays the groundwork for their identification and differentiation. As shown in Figure 6D, the HOMO and LUMO of L-Glu, D-Glu, and ZIF-8 are (−6.8785 eV, −0.5448 eV), (−6.8461 eV, −0.2800 eV), and (−4.9748 eV, −4.2297 eV), respectively. The E_gap_ of L-Glu and D-Glu was calculated to be 6.34 eV and 6.57 eV, respectively. A smaller E_gap_ of L-Glu indicates more reactive molecule, while the larger one of D-Glu suggests more stable molecule [45]. The energy level difference between the HOMO of ZIF-8 and the LUMO of L-Glu is 4.43 eV, whereas it is 4.69 eV for D-Glu. The smaller energy gap implies that electrons can more readily transfer from the HOMO of ZIF-8 to the LUMO of L-Glu, resulting in stronger interactions between them [45]. In contrast, a larger energy gap suggests that electron transfer from the HOMO of ZIF-8 to the LUMO of D-Glu is more challenging, leading to a weaker interaction. Additionally, the computational results indicate that L-Glu (excited state: 5.2231 eV, 237.38 nm) has lower excitation energy compared with D-Glu (excited state: 5.2863 eV, 234.54 nm). This suggests that electrons in L-Glu are more prone to transfer and exhibit stronger interactions with the ZIF-8 material [46]. Additionally, the analysis through DFT calculations reveals that the binding energy of ZIF-8 with L-Glu is 39.55 kcal/mol, while with D-Glu is 70.95 kcal/mol (Figure 6E). The lower the binding energy, the stronger the intermolecular interactions [47]. Consequently, the interaction between ZIF-8 and L-Glu is stronger than that with D-Glu. These results explain the experimental observation that Mn_x_O_y_ NWs@ZIF-8-RD can discriminate Glu enantiomers. It is important to note that the present DFT model is specifically designed to elucidate the chiral recognition capability of the ZIF-8 adsorbent. A more comprehensive model that explicitly includes the Mn_x_O_y_ catalytic interface and solvation effects will be the focus of future study, more computationally intensive investigations to provide a complete picture of the integrated catalytic cycle.

## 4. Conclusions

In summary, this study successfully synthesized a material of Mn_x_O_y_ NWs@ZIF-8-RD with Glu OXD- and POD-like activities, which can specifically recognize L/D-Glu enantiomers with high efficiency. The chiral recognition mechanism of Mn_x_O_y_ NWs@ZIF-8-RD towards L/D-Glu was confirmed through experiments and DFT calculation. Additionally, the enzyme-like activity of Mn_x_O_y_ NWs@ZIF-8-RD can achieve spiked detection of Glu in complex biological samples, showcasing excellent selectivity and resistance to interference. The innovations of this study are as follows: (1) The material of Mn_x_O_y_ NWs@ZIF-8-RD was synthesized via mild conditions, with varying sizes and possessing multiple enzyme-like activities with high catalytic efficiency (*V*_max_ value of H_2_O_2_: 840.52 × 10^−8^ M·S^−1^). (2) Mn_x_O_y_ NWs@ZIF-8-RD can specifically recognize and quantitatively detect Glu enantiomers. (3) A combination of experimental and theoretical methods elucidated the chiral recognition mechanism, offering a deeper understanding of the interaction between materials and molecules, which can guide the design of more efficient chiral recognition systems. (4) The key factors influencing the synthesis of specific sizes and morphologies of ZIF-8 were investigated, providing a reference for subsequent studies. (5) The successfully developed method for identifying and detecting Glu enantiomers offers methodological insights for the recognition of AAs enantiomers.

## Figures and Tables

**Table 1:** Apparent Michaelis-Menten constant (*K*_m_) and the maximum reaction rate (*V*_max_) of reported enzyme mimics.

Enzyme Mimics	H_2_O_2_		OPD		Specific Activity (μmol·min^−1^·mg^−1^)	TOF (min)	Ref.
	*K*_m_ (mM)	*V*_max_ (10^−8^ M/S)	*K*_m_ (mM)	*V*_max_ (10^−8^ M/S)			
CDs (Colorimetry)	0.7568	1.086	6.81	0.76	–	–	[28]
Cu_23_ NC (Colorimetry)	1.0	0.00467	1.09	0.02	–	12.05	[29]
HRP (Colorimetry)	0.15	0.077	1.800	0.120	–	1.32	[30]
MIL-3DG-75 (Fluorometry)	0.029	0.011	49.5	18	–	–	[31]
Cu-CDs (Fluorometry)	–	–	0.588	4.256	–	–	[32]
Hemin-histamine pair	1.37	5.29	1.63 (ABTS)	11.70 (ABTS)	36.45	1.758	[33]
Hemin	–	–	2.33 (ABTS)	0.97 (ABTS)	0.63	0.144	
Mn_x_O_y_ NWs@ZIF-8-RD (Fluorometry)	1.066	840.52	0.1863	64.76	1.284 (Composite material); 24.69 (Mn total); 17.30 (Mn_x_O_y_)	1.358	This work

## Data Availability

Data are contained within the article and supplementary materials.

## Supplementary Materials

The following supporting information can be downloaded at https://www.mdpi.com/article/10.3390/bios15120771/s1, Figure S1: Characterizations of Mn_x_O_y_ NWs. XPS full spectra (A); C 1s (B), N 1s (C), O 1s (D), and Mn 2p (E) spectra; FT-IR spectra (F); TGA results (G); XRD results (H) of Mn_x_O_y_ NWs; Figure S2: The effect of MnO_4_^−^ concentrations on the synthesis of Mn_x_O_y_ NWs@ZIF-8-RD. ESEM images of 1.6 M (A), 1.4 M (B), 1.2 M (C), 1.0 M (D), and 0.8 M (E); XRD results (F); FT-IR spectra (G); fluorescence intensity for Glu enantiomer recognition (H); Figure S3: The effect of Zn(II)/Hmim molar ratio on the synthesis of Mn_x_O_y_ NWs@ZIF-8-RD. ESEM images of Mn_x_O_y_ NWs@ZIF-8-RD synthesized with Zn(II)/Hmim molar ratios of 1:2 (A), 1:3 (B), 2:1 (C), 2:3 (D), 3:1 (E), and 3:2 (F); and their XRD patterns (G), FT-IR spectra (H), the activity for Glu enantiomer recognition (I); Figure S4: The effect of synthesis temperature on the prepared materials. ESEM images of Mn_x_O_y_ NWs@ZIF-8-RD synthesized at 30 °C (A) and 60 °C (B). XRD results (C); FT-IR spectra (D); and the fluorescence intensity for Glu enantiomer recognition (E) of Mn_x_O_y_ NWs@ZIF-8-RD; Figure S5: The effects of synthesis time on the Mn_x_O_y_ NWs@ZIF-8-RD. ESEM images of 30 min (A), 60 min (B), 90 min (C), 120 min (D), and 150 min (E); XRD results (F); FT-IR spectra (G); and the fluorescence intensity for Glu enantiomer recognition (H) of Mn_x_O_y_ NWs@ZIF-8-RD; Figure S6: The fluorescence intensity for Glu enantiomer recognition by Mn_x_O_y_ NWs@ZIF-8-RD at buffer pH of 4 (A), 6.5 (B), and 9 (C). Schematic diagram of enzyme activity regulation (D). The effect of buffer pH (E), reaction temperature (F), material dilution ratio (G), OPD concentration (H), and reaction time (I) on Glu enantiomer recognition; Figure S7: The linear relationships between L/D-Glu concentrations and fluorescence intensity (A,B). The storage stability of Mn_x_O_y_ NWs@ZIF-8-RD (C) for Glu enantiomer recognition. Effects of various free radical scavengers on the catalysis of H_2_O_2_ + OPD by Mn_x_O_y_ NWs@ZIF-8-RD (D). Fluorescence spectra of Mn_x_O_y_ NWs@ZIF-8-RD + H_2_O_2_ + OPD reaction solution containing BQ (E) with varying concentrations from 0.021 to 0.86 mM. Direct EPR evidence of $O_{2}^{•-}$ production via DMPO trapping (F). The mechanism of materials catalyzed Glu reaction (G). The influence of other interfering substances on the reaction system (H). Differences in activity (I) and morphology between material batches (J–L); Figure S8: Identification of H_2_O_2_ through SOD catalyzed reaction (A). Identification of α-keto acids by LC-MS (B). Figure S9: The ZIF-8’ precise spatial configuration obtained from the Cambridge Structural Database (CSD); Table S1: The distribution of elements in the Mn_x_O_y_ NWs@ZIF-8-RD; Table S2: Comparison of reported sensor platforms for Glu detection; Table S3: Detection of L/D-Glu in rabbit plasma; Table S4: Chemical parameters of L-Glu, D-Glu, and ZIF-8. References [3,48,49,50,51,52,53,54] are cited in the Supplementary Materials.
