# Supplementary material for: Mn Oxide Nanowire/ZIF-8 Composites with Multiple Enzyme-like Activities for Enantioselective Glutamate Sensing
Source: Biosensors (Basel). 2025 Nov 25;15(12):771. doi: 10.3390/bios15120771 (PMC12730338; doi:10.3390/bios15120771)
Supplement: Supplementary file 1 [file biosensors-15-00771-s001.zip › biosensors-3946830-supplementary.pdf]

# Supplementary Materials

## Mn Oxide Nanowire/ZIF-8 Composites with Multiple Enzyme-like Activities for Enantioselective Glutamate Sensing

**Guo-Ying Chen, Mao-Ling Luo, Jing-Jing Dai and Feng-Qing Yang ***

Department of Pharmaceutical Engineering, School of Chemistry and Chemical Engineering, Chongqing University, Chongqing 401331, China; 20221801017@stu.cqu.edu.cn (G.-Y.C.); 201718021124@cqu.edu.cn (M.-L.L.); 202018021119@cqu.edu.cn (J.-J.D.)

* Correspondence: fengqingyang@cqu.edu.cn

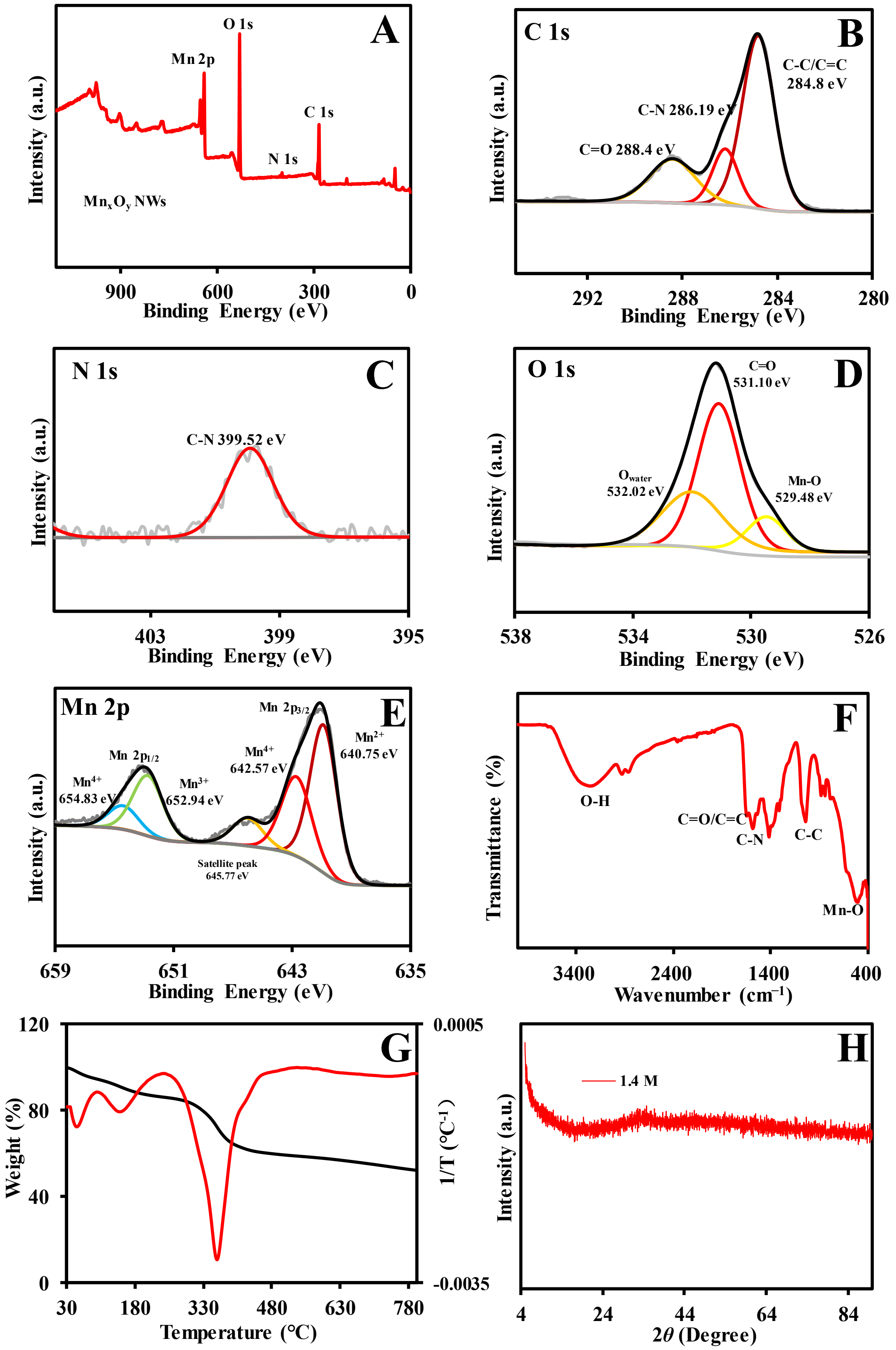


**Figure S1.** Characterizations of $Mn_xO_y$ NWs. XPS full spectra (A); C 1s (B), N 1s (C), O 1s (D), and Mn 2p (E) spectra; FT-IR spectra (F); TGA results (G); XRD results (H) of $Mn_xO_y$ NWs.

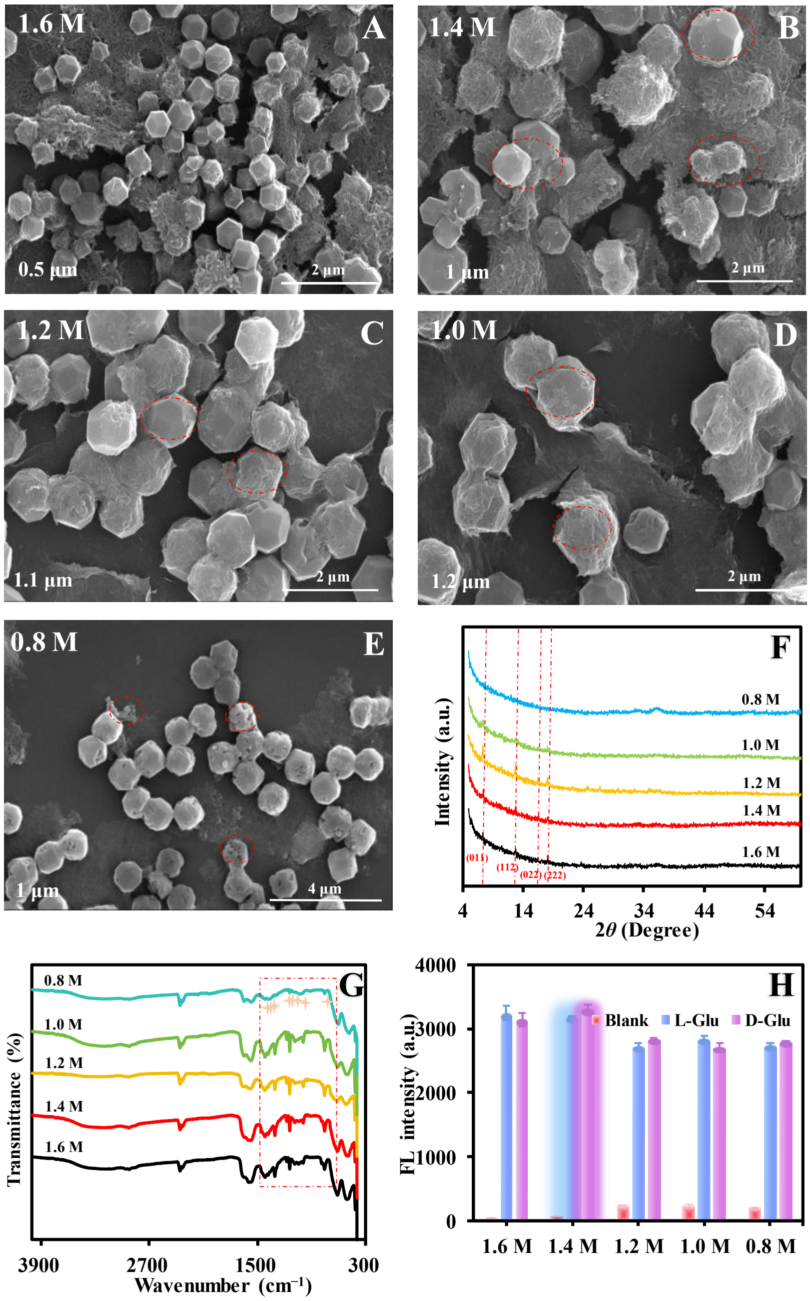


**Figure S2.** The effect of $MnO_4^-$ concentrations on the synthesis of $Mn_xO_y$ NWs@ZIF-8-RD. ESEM images of 1.6 M (A), 1.4 M (B), 1.2 M (C), 1.0 M (D), and 0.8 M (E); XRD results (F); FT-IR spectra (G); fluorescence intensity for Glu enantiomer recognition (H).

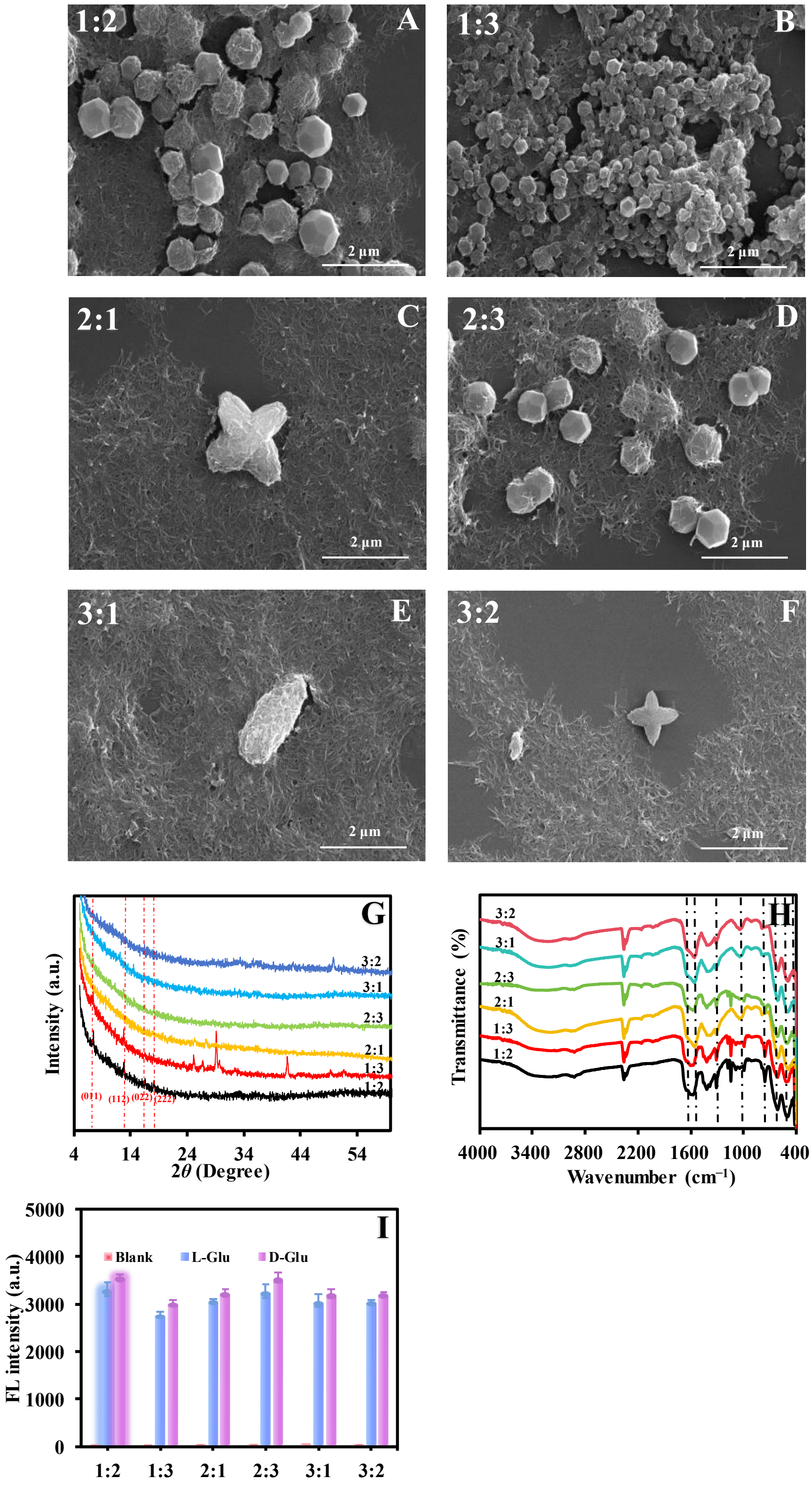


**Figure S3.** The effect of Zn(II)/Hmim molar ratio on the synthesis of $Mn_xO_y$ NWs@ZIF-8-RD. ESEM images of $Mn_xO_y$ NWs@ZIF-8-RD synthesized with Zn(II)/Hmim molar ratios of 1:2 (A), 1:3 (B), 2:1 (C), 2:3 (D), 3:1 (E), and 3:2 (F); and their XRD patterns (G), FT-IR spectra (H), the activity for Glu enantiomer recognition (I).

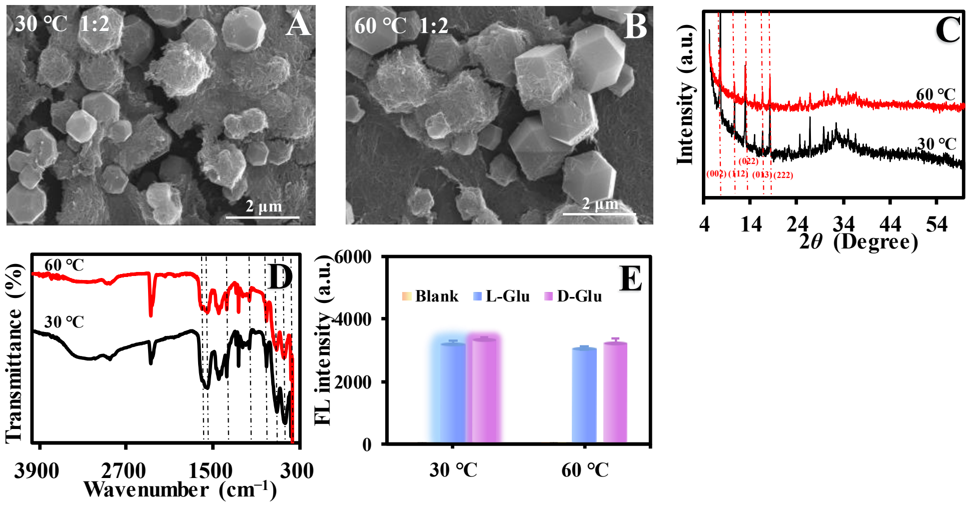


**Figure S4.** The effect of synthesis temperature on the prepared materials. ESEM images of $Mn_xO_y$ NWs@ZIF-8-RD synthesized at 30 °C (A) and 60 °C (B). XRD results (C); FT-IR spectra (D); and the fluorescence intensity for Glu enantiomer recognition (E) of $Mn_xO_y$ NWs@ZIF-8-RD.

The ESEM results illustrate distinct morphological differences in the $Mn_xO_y$ NWs@ZIF-8-RD synthesized at 30 °C and 60 °C (Figure S4A and S4B). At the temperature of 30 °C, the MnxOy NWs@ZIF-8-RD forms polyhedral structures, including dodecahedrons, tetrakaidecahedrons, and hexadecahedrons, with sizes ranging from 0.5 to 1.1 µm. In contrast, at the higher temperature of 60 °C, the material predominantly exhibits a rhombic dodecahedral morphology, with sizes between 0.8 and 1.5 µm. Both $Mn_xO_y$ NWs@ZIF-8-RD synthesized at 30 °C and 60 °C display characteristic diffraction peaks corresponding to the crystal planes of ZIF-8 (0 0 2, 1 1 2, 0 2 2, 0 1 3, 2 2 2) (Figure S4C). The intensity of these peaks is slightly higher in the material synthesized at 30 °C, likely due to the changes in the crystal structure, such as phase transitions or alterations in lattice parameters, which can affect the position and intensity of the diffraction peaks [48]. FT-IR spectra (Figure S4D) confirm that $Mn_xO_y$ NWs@ZIF-8-RD retains the vibrational peaks of functional groups of ZIF-8 and $Mn_xO_y$ NWs, including the imidazole rings, Zn–N, and Mn–O bonds. In terms of Glu enantiomers identification, $Mn_xO_y$ NWs@ZIF-8-RD synthesized at both temperatures show no significant difference in activity (Figure S4E). Nonetheless, considering the favorable stability and potential for subsequent applications under milder conditions, a synthesis temperature of 30 °C was selected for the synthesis of $Mn_xO_y$ NWs@ZIF-8-RD in further experiments.

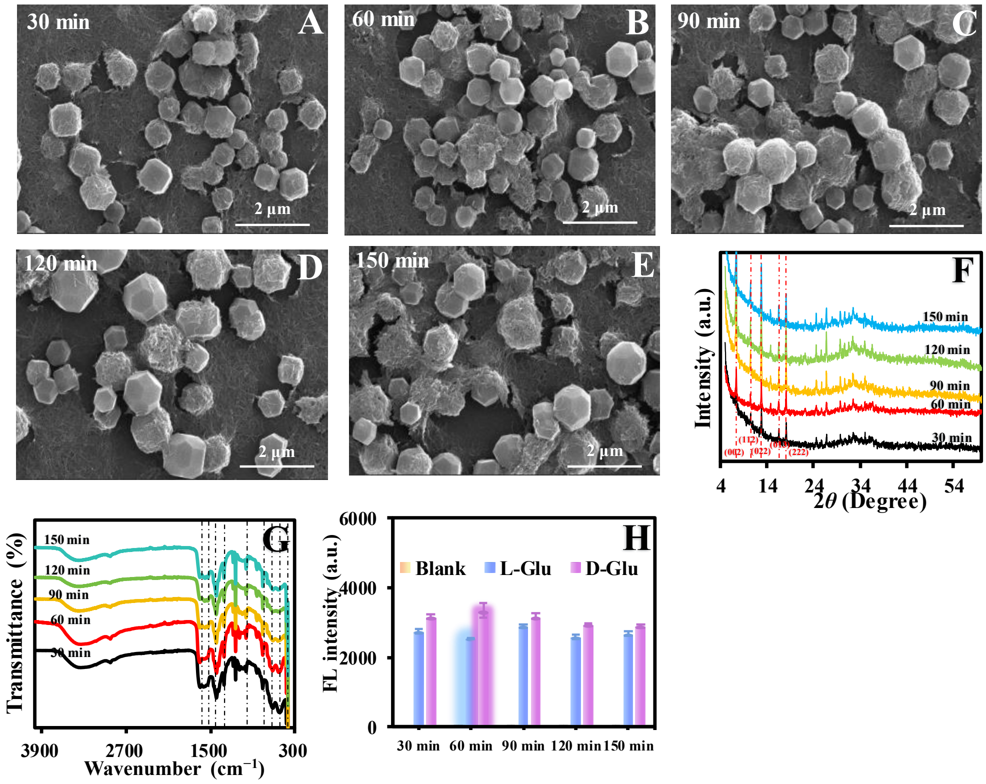


**Figure S5.** The effects of synthesis time on the $Mn_xO_y$ NWs@ZIF-8-RD. ESEM images of 30 min (A), 60 min (B), 90 min (C), 120 min (D), and 150 min (E); XRD results (F); FT-IR spectra (G); and the fluorescence intensity for Glu enantiomer recognition (H) of $Mn_xO_y$ NWs@ZIF-8-RD.

$Mn_xO_y$ NWs@ZIF-8-RD exhibits diverse morphologies and size distributions at different synthesis time (30, 60, 90, 120, and 150 min) (Figure S5). As synthesis time increased, $Mn_xO_y$ NWs@ZIF-8-RD predominantly forms rhombic dodecahedral structure, with the concentrated sizes distribution mainly ranging from 0.6 to 0.8 μm (Figure S5A–E). Notably, at the synthesis time of 60 min, $Mn_xO_y$ NWs@ZIF-8-RD achieves a relatively uniform size (Figure S5B). Further analysis reveals that across various reaction time, $Mn_xO_y$ NWs@ZIF-8-RD consistently displays characteristic diffraction peaks corresponding to the crystal planes (0 0 2, 1 1 2, 0 2 2, 0 1 3, 2 2 2) of ZIF-8 in the XRD spectra (Figure S5F), and the FT-IR spectra consistently show vibrational peaks of functional groups of ZIF-8 and $Mn_xO_y$ NWs (Figure S5G). The intensity and position of these peaks (Zn–N/Mn–O, and imidazole ring) remain relatively unchanged across different reaction time, robustly demonstrating the stability of $Mn_xO_y$ NWs@ZIF-8-RD's crystal structure. Additionally, for identifying Glu enantiomers (Figure S5H), the highest fluorescence ratio and fluorescence intensity values were obtained at the synthesis time of 60 min, which was selected for subsequent experiments.

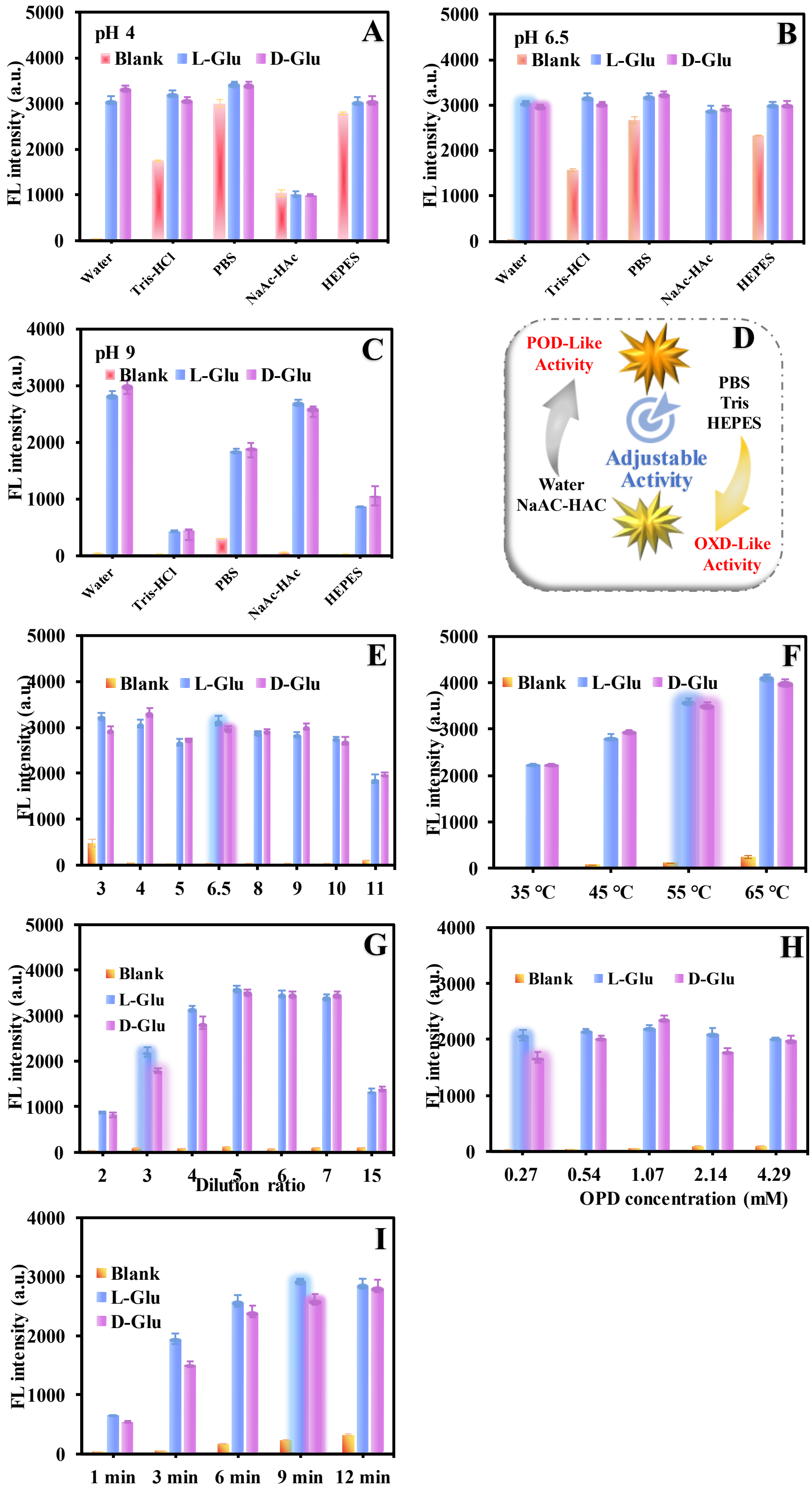


**Figure S6.** The fluorescence intensity for Glu enantiomer recognition by $Mn_xO_y$ NWs@ZIF-8-RD at buffer pH of 4 (A), 6.5 (B), and 9 (C). Schematic diagram of enzyme activity regulation (D). The effect of buffer pH (E), reaction temperature (F), material dilution ratio (G), OPD concentration (H), and reaction time (I) on Glu enantiomer recognition.

The type of buffer solution and pH value play a crucial role in enzymatic catalysis. Proper selection of the buffer and pH not only optimizes the reaction conditions but also mimics the physiological environment, reducing non-specific effects, thereby enhancing the accuracy and reliability of the experiment [49, 50]. As depicted in Figure S6, the Glu enantiomers recognition by using $Mn_xO_y$ NWs@ZIF-8-RD (Figure S6A–S6C), across various buffer solutions, including water, Tris-HCl, PBS, sodium acetate-acetic acid (NaAC-HAC), and HEPES solutions, under acidic (pH 4), neutral (pH 6.5), and alkaline (pH 9) conditions were explored, respectively. The results uncover distinct enzymatic catalytic activity for $Mn_xO_y$ NWs@ZIF-8-RD under different buffer and pH conditions. Notably, in water and NaAC-HAC solutions, all materials displayed superior POD-like activity, facilitating the production of $H_2O_2$ from Glu and subsequently catalyzing the oxidation of OPD to ox-OPD. In Tris-HCl, PBS, and HEPES solutions, the materials exhibited notable OXD-like activity, directly catalyzing the oxidation of OPD (Figure S6D). While OXD activity is typically enhanced under acidic conditions and diminished under neutral and alkaline ones [51], the material of $Mn_xO_y$ NWs@ZIF-8-RD maintained relatively high activity under neutral conditions (Figure S6B), demonstrating the potential application in practical samples based on its OXD enzyme activity, such as in biological matrices. For Glu enantiomers identification, the POD and OXD-like activities of $Mn_xO_y$ NWs@ZIF-8-RD were utilized. Therefore, $Mn_xO_y$ NWs@ZIF-8-RD in aqueous solutions was used to identify Glu enantiomers. It shows versatile enzymatic catalytic activities under varying buffer and pH conditions, offering potential for diverse applications. Based on the experimental outcomes for identifying Glu enantiomers in aqueous solutions of $Mn_xO_y$ NWs@ZIF-8-RD, the variation in solution pH from 3 to 11 significantly affects the stability of detection (Figure S6E). The material maintains stability across a pH range from 5 to 10. Considering practical sample applications, a pH value of 6.5 was chosen for subsequent experiments. The fluorescence intensity of the material increases with the reaction temperature and stabilizes after 55 °C (Figure S6F), which was used for further experiments. Material concentration substantially impacts the chemical reaction rates. Thus, optimizing material concentration is key for obtaining efficient and stable reaction conditions. The fluorescence intensity changed with dilution factors ranging from 2 to 15 of the materials $Mn_xO_y$ NWs@ZIF-8-RD (Figure S6G). Considering the differences in catalytic activity and enantiomer recognition, the dilution ratio of $Mn_xO_y$ NWs@ZIF-8-RD at 3

demonstrated a marked distinction in the ability to discern Glu enantiomers, which was chosen for subsequent Glu enantiomer identification. The effect of substrate concentration (OPD, initial concentration: 0.63–10.00 mM, final concentration: 0.27–4.29 mM) was examined. The fluorescence intensity values (Figure S6H) remain relatively stable as the substrate concentration changes, which suggest that the material has a strong affinity for the substrate, effectively binding and catalyzing the reaction even at low substrate concentrations. Therefore, the OPD concentration of 0.27 mM was selected for further study. The fluorescence intensity increases with reaction time and stabilizes after 9 min (Figure S6I). Therefore, the reaction time of 9 min was chosen for subsequent experiments.

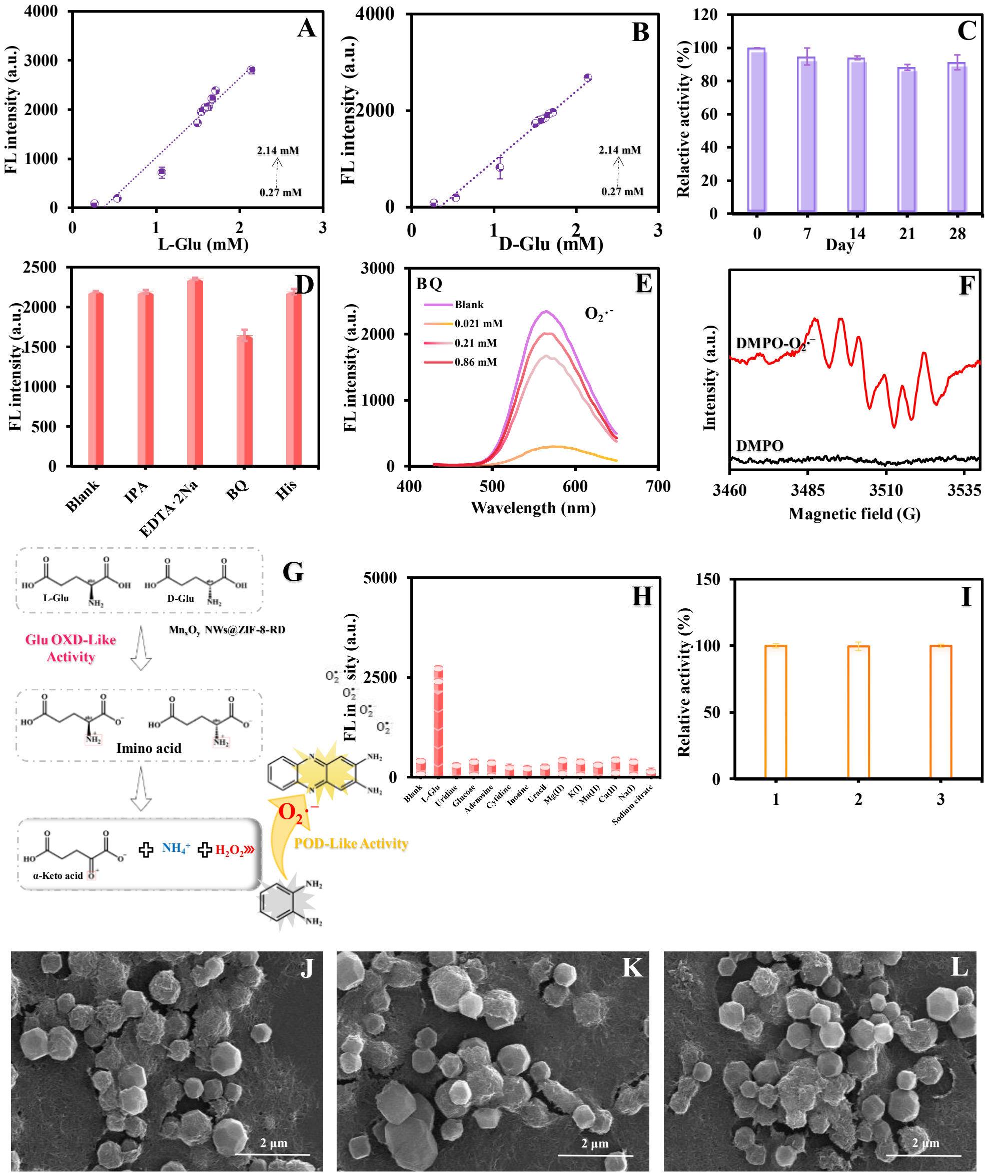


**Figure S7.** The linear relationships between L/D-Glu concentrations and fluorescence intensity (A, B). The storage stability of $Mn_xO_y$ NWs@ZIF-8-RD (C) for Glu enantiomer recognition. Effects of various free radical scavengers on the catalysis of $H_2O_2$ + OPD by $Mn_xO_y$ NWs@ZIF-8-RD (D). Fluorescence spectra of $Mn_xO_y$ NWs@ZIF-8-RD + $H_2O_2$ + OPD reaction solution containing BQ (E) with varying concentrations from 0.021 to 0.86 mM. Direct EPR evidence of $O_2^{\bullet -}$ production via DMPO trapping (F). The mechanism of materials catalyzed Glu reaction (G). The influence of other interfering substances on the reaction system (H). Differences in activity (I) and morphology between material batches (J–L).

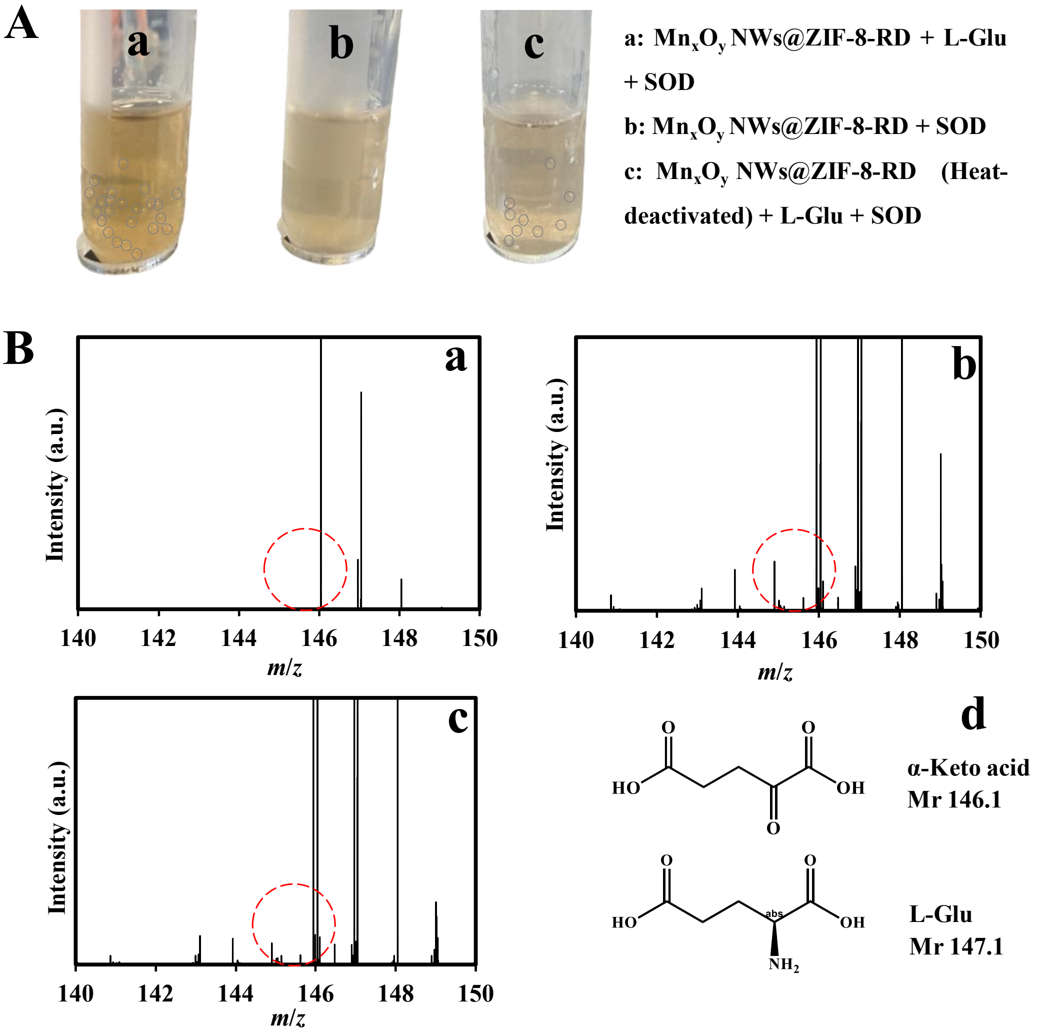


**Figure S8.** Identification of $H_2O_2$ through SOD catalyzed reaction (A). Identification of $\alpha$-keto acids by LC-MS (B). For B, a: L-Glu, b: $Mn_xO_y$ NWs@ZIF-8-RD + L-Glu, c: $Mn_xO_y$ NWs@ZIF-8-RD (heat-deactivated) + L-Glu, d: the structural formula of L-Glu and $\alpha$-keto acids.

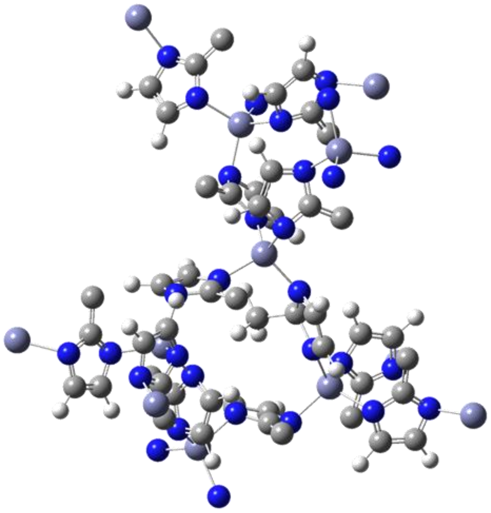

**Figure S9.** The ZIF-8′ precise spatial configuration obtained from the Cambridge Structural Database (CSD).

**Table S1**. The distribution of elements in the $Mn_xO_y$ NWs@ZIF-8-RD.

| Elements | Atomic Fraction (%) | Mass Fraction (%) |
|---|---|---|
| C | 62.58 | 6.61 |
| N | 20.04 | 4.49 |
| O | 6.35 | 5.53 |
| Mn | 1.74 | 5.20 |
| Zn | 9.29 | 33.07 |

**Table S2**. Comparison of reported sensor platforms for Glu detection.

| Materials | Linear range (mM) | LOD (μM) | Ref. |
|---|---|---|---|
| PDMA-GCE (Electrochemical) | 0.5–15 | 110 (L); 260 (D) | [52] |
| Chiral gSiNPs (Fluorometry) | 0.01–8 | 3.1 (D) | [3] |
| PDADs (Fluorometry) | 0–100 | 0.12 | [53] |
| Gldh bacteria and MWNTs (Electrochemical) | 10–1/2–10 | 2 | [54] |
| $Mn_xO_y$ NWs@ZIF-8-TH (Fluorometry) | 0.27–2.14 | 8.29 (L); 12.76 (D) | This work |

**Table S3**. Detection of L/D-Glu in rabbit plasma.

| Sample | Added (mM) | Founded (mM) | Recovery (%) | RSD ($n$ = 3) |
|---|---|---|---|---|
| L-Glu | 0 | – | – | – |
| | 0.43 | 0.46 | 106.2 | 3.3 |
| | 0.64 | 0.71 | 110.9 | 1.0 |
| | 0.86 | 0.91 | 105.9 | 3.3 |
| D-Glu | 0.43 | 0.40 | 93.0 | 3.2 |
| | 0.64 | 0.66 | 103.1 | 2.5 |
| | 0.86 | 0.87 | 101.2 | 2.5 |

**Table S4**. Chemical parameters of L-Glu, D-Glu, and ZIF-8.

| | η | μ | χ | σ | ω | S |
|---|---|---|---|---|---|---|
| L-Glu | 3.17 | -3.71 | 3.71 | 0.3154 | 2.1710 | 0.1577 |
| D-Glu | 3.285 | -3.565 | 3.565 | 0.3044 | 1.9344 | 0.1522 |
| ZIF-8 | 0.37 | -4.6 | 4.6 | 2.7027 | 28.5946 | 1.3514 |

**η**, total hardness; **μ**, chemical potential; **χ**, electronegativity; **σ**, absolute softness; **ω**, electrophilicity index; **S**, total softness.
